# Medical therapy versus radiofrequency endometrial ablation in the initial treatment of heavy menstrual bleeding (iTOM Trial): A clinical and economic analysis

**DOI:** 10.1371/journal.pone.0188176

**Published:** 2017-11-15

**Authors:** Abimbola O. Famuyide, Shannon K. Laughlin-Tommaso, Sherif A. Shazly, Kirsten Hall Long, Daniel M. Breitkopf, Amy L. Weaver, Michaela E. McGree, Sherif A. El-Nashar, Maureen A. Lemens, Matthew R. Hopkins

**Affiliations:** 1 Minimally Invasive Gynecologic Surgery, Department of Obstetrics and Gynecology, Mayo Clinic, Rochester, Minnesota, United States of America; 2 K. Long Health Economics Consulting LLC, St. Paul, Minnesota, United States of America; 3 Division of Biomedical Statistics and Informatics, Department of Health Sciences Research, Mayo Clinic, Rochester, Minnesota, United States of America; 4 Division of Female Pelvic Medicine and Reconstructive Surgery, University Hospitals, Cleveland, Ohio, United States of America; Duke University, UNITED STATES

## Abstract

**Background:**

Radiofrequency endometrial ablation (REA) is currently a second line treatment in women with heavy menstrual bleeding (MHB) if medical therapy (MTP) is contraindicated or unsatisfactory. Our objective is to compare the effectiveness and cost burden of MTP and REA in the initial treatment of HMB.

**Methods:**

We performed a randomized trial at Mayo Clinic Rochester, Minnesota. The planned sample size was 60 patients per arm. A total of 67 women with HMB were randomly allocated to receive oral contraceptive pills (Nordette ^®^) or Naproxen (Naprosyn^®^) (n = 33) or REA (n = 34). Primary 12-month outcome measures included menstrual blood loss using pictorial blood loss assessment chart (PBLAC), patients’ satisfaction, and Menorrhagia Multi-Attribute Scale (MMAS). Secondary outcomes were total costs including direct medical and indirect costs associated with healthcare use, patient out-of-pocket costs, and lost work days and activity limitations over 12 months.

**Results:**

Compared to MTP arm, women who received REA had a significantly lower PBLAC score (median [Interquartile range, IQR]: 0 [0–4] vs. 15 [0–131], p = 0.003), higher satisfaction rates (96.8%vs.63.2%, p = 0.003) and higher MMAS (median [IQR]: 100 [100–100] vs. 100 [87–100], p = 0.12) at 12 months. Direct medical costs were higher for REA ($5,331vs.$2,901, 95% confidence interval (CI) of mean difference:$727,$4,852), however, when indirect costs are included, the difference did not reach statistical significance ($5,469 vs. $3,869, 95% CI of mean difference:-$339, $4,089).

**Conclusion:**

For women with heavy menstrual bleeding, initial radiofrequency endometrial ablation compared to medical therapy offered superior reduction in menstrual blood loss and improvement in quality of life without significant differences in total costs of care.

**Clinical trial registration:**

NCT01165307.

## Introduction

Heavy menstrual bleeding (HMB) is a common gynecological condition afflicting women of reproductive age, often resulting in physical, psychological and social incapacitation. [1, 2] Both the American College of Obstetricians and Gynecologists and the National Institute for Health and Care Excellence in the United Kingdom recommend medical therapy (MTP) as initial treatment for women whose excessive menstrual blood loss (MBL) adversely impacts their quality of life.[3–5] Traditionally, MTP consists of combined monophasic oral contraceptive pills (OCP), oral or injectable progesterone, non-steroidal anti-inflammatory agents (NSAID), and more recently tranexamic acid and levonorgestrel intrauterine systems (LNG-IUS) which gained US Food and Drug Administration (FDA) approval for use in heavy menstrual bleeding.

Hysterectomy had been the mainstay of alternative treatment option for women who found medical therapy ineffective or were intolerant of side-effects, until the first non-hysteroscopy dependent device for endometrial ablation gained FDA approval for use in 1997. These devices are now second line treatment choice after MTP has failed. The radiofrequency endometrial ablation device (REA, Novasure, Hologic^®^) is one of six devices in use that directly delivers energy to the endometrium and is highly effective with minimal complications when used in appropriately selected patients. [6, 7]

Given the variation in effectiveness, tolerance and appeal of MTP[8–11], and likely cost differential between initial treatment options, we performed a cost-consequences analysis (CCA) to evaluate the effectiveness and economic impact of treating women with HMB without future fertility desire, with MTP or REA as initial therapy. The study interventions included 2 of the commonly prescribed therapeutic agents- oral contraceptive pills and a non-steroidal anti-inflammatory agent to reflect the practical options women and their providers have in treating HMB.

## Materials and methods

We performed a non-blinded randomized control trial at Mayo Clinic Rochester, MN, US, a tertiary academic center in the upper Midwest. Our trial was approved by Mayo Clinic, Rochester institutional review board (IRB) on February 26^th^ 2009 and the first patient was recruited August 5^th^ 2009 and the last on September 9^th^ 2012. At the time of first enrollment, we were not aware of the requirement of registering trials on clinical trials.gov but did so in July 2010 once the policy was brought to our attention. Authors confirm that all ongoing and related trials for this drug/intervention are registered. Women who sought first time treatment for HMB were asked to participate in the trial if they met the inclusion criteria, including, ages 30–55, subjective symptom of excessive menstrual bleeding, at least one normal Pap test within previous 3 years, prior history of permanent sterilization, or use of a reliable non-hormonal contraceptive or reliance on partner’s vasectomy. Women were excluded from enrollment if they did not meet criteria for REA or had contraindication to both OCP and NSAID.[12] An informed written consent was obtained from all participants. All women were evaluated by office flexible hysteroscopy as per previous publication [13], Pipelle endometrial biopsy, and a Pap test if none was documented in the preceding 12 months.

All consented women had a one month lead-in period, during which baseline demographic and clinical information were obtained, including assessment of menstrual blood loss using pictorial blood loss assessment chart (PBLAC), dysmenorrhea, and premenstrual symptoms diary. Following the lead-in period, women were randomized to MTP or REA using a dynamic allocation method to ensure balance between the treatment groups based on stratification attributes; age (<45 years, ≥45 years), parity (<2, ≥2) and body mass index (BMI<30 kg/m^2^, ≥30 kg/m^2^). The randomization assignment for each patient was obtained by entering the patient’s stratification levels into a web-based computer application. Women who were allocated to MTP received a prescription for 30 microgram estradiol/150 mcg levonorgestrel monophasic oral contraceptive pills (Nordette^®^). Using this formulation, Fraser et al (1991) showed a 43% reduction in MBL in women with HMB.[14] Women were instructed to administer the pills orally, starting five days after the start of menstrual blood flow continuing cyclically, thus allowing for withdrawal bleeding after the 21 day pill cycle. For those unable to tolerate or were unwilling to accept OCP, naproxen sodium (Naprosyn^®^) was prescribed: 500 mg with onset of menses, followed by 250 mg three times daily for the duration of menses or a maximum of five days. This regimen showed no difference in blood loss reduction when compared to Nordette.[14] Medications were issued to cover an initial period of 3 months, with re-fills up to 1 year.

Women allocated to the REA arm received therapy within four weeks of randomization and the procedure was performed by one of four surgeons (AOF, SKL, DMB, and MRH) in an ambulatory surgery unit under conscious sedation. The procedure was performed per manufacturer’s instructions.[12] All perioperative and intraoperative adverse events including cervical laceration, uterine perforation, and bleeding were recorded.

Primary endpoints included bleeding score as noted on the PBLAC collected at baseline and 12 months. Women were allowed to use their own sanitary products. PBLAC scores in excess of 150 are considered abnormal and correlate with MBL as determined by alkaline hematin test.[15, 16] Bleeding pattern was collated and analyzed using the World Health Organization (WHO) criteria for clinically important bleeding patterns. Patient satisfaction was ascertained by asking study participants to choose from one of four categories relating to their general satisfaction with treatment: totally satisfied, generally satisfied, acceptable improvement in symptoms, or unacceptable treatment. General health-related quality of life was assessed using SF-12^®^ and disease-specific quality of life was evaluated using Menorrhagia Multi-Attribute Scale (MMAS).[17] We have previously validated both tools in a cohort of women with HMB [18]. A premenstrual diary was kept and documented by each patient at 3-, 6- and 12-month visits. Pain or dysmenorrhea was assessed by 0–10 visual analogue scale.

Economic analysis was conducted from a limited societal perspective and included direct and indirect costs of care associated with medical compared with surgical treatment but did not consider spillover costs outside of the healthcare sector. Total direct medical costs included procedural, post-procedural care, outpatient and inpatient visits, and inpatient medication costs incurred during 12 months post-enrollment and were tracked using administrative data from the Mayo Clinic Cost Database. Further, because of well-known discrepancies between billed charges and true resource use, utilization was valued by grouping services into the Medicare Part A and Part B classification: Part A billed charges (hospital-billed services and procedures) were adjusted using hospital cost-to-charge ratios at the departmental level and wage indexes. Medicare Part B items (primarily physician-billed services) were valued using national average Medicare reimbursement rates by Current Procedural Terminology (CPT-4) code.

Indirect costs of care were also assessed and included costs associated with sanitary product use, days with limited activity, and lost work days due to illness. Data on sanitary product use was collected at 3-, 6- and 12-month visits and a 9-month phone interview and were valued using national average sales prices. Information on reduced capacity (limited activity and lost work days) was assessed in menstrual diaries collected at 3, 6- and 12 months. Patient days with limited capacity days were valued using standard human capital methods based on gender-specific median hourly wage rates from the Bureau of Labor Statistics Current Population Survey [19].

Using PBLAC scores at 12 months as our primary end-point, we estimated 120 patients per arm would be required to detect an effect size (natural log of the ratio of the two means) of 0.40 between the treatment arms (assuming a mean PBLAC score at 12 months of 49 in the REA arm and 73 in the MTP arm) with 80% power, type 1 error of 5%, and an anticipated 20% loss to follow-up. These estimates were based on prior studies that showed 50% reduction in PBLAC score in the endometrial ablation arm and 30% reduction in those who received MTP [20, 21]. By February 2011, as a result of a lower than anticipated recruitment, we revised our target to 60 patients per arm with a power of 80% to detect an effect size of 0.57. Assuming a mean PBLAC score at 12 months of 49 in the REA arm, this effect size would equate to a mean PBLAC score at 12 months of 87 in the MTP arm.

Standard descriptive statistics were generated for measurements at baseline and 12 months, including mean, median, standard deviation (SD), and interquartile range (IQR) for continuous variables and counts and percentages for categorical variables. Categorical variables were compared between the two treatment arms using the chi-square test or Fisher’s exact test as appropriate. In addition, for each 12-month continuously scaled clinical outcome measure, separate analysis of covariance (ANOCOVA) models were fit to compare the measures between the two treatment arms after adjusting for baseline levels. Patient satisfaction scores were rated on a 4-point ordinal scale and compared using Wilcoxon rank-sum tests. The treatment failure rate for each arm within the first 12 months was estimated using the Kaplan-Meier method and compared between arms using the log-rank test. Observed costs were compared using non-parametric bootstrapped CIs of mean differences, accounting for the skewed nature of cost data. [22, 23] Crossover was allowed after three months following enrollment, but all analysis were based on the intention to treat principle. A secondary per protocol analysis was also performed to evaluate differences between MTP only, MTP and endometrial ablation, and REA. All calculated P values were two-sided and values less than 0.05 were considered statistically significant. Statistical analyses were performed using the SAS version 9.3 software package (SAS Institute, Inc.; Cary, NC).

## Results

**Fig 1:**
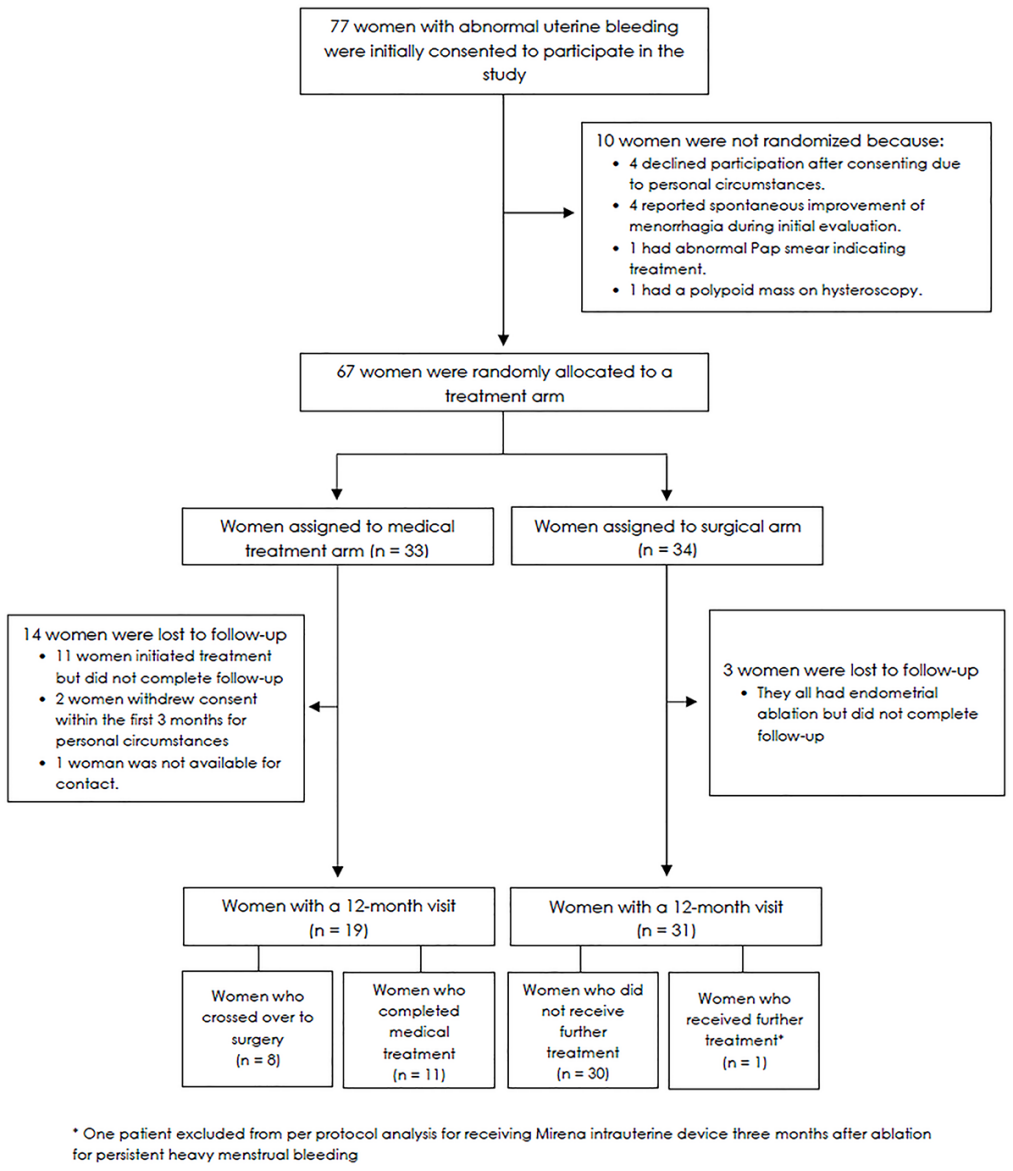
Study flow chart.

Over a period spanning 3.5 years, 631 patients who presented with HMB were screened. Of these, documentation was available on 471 women found ineligible for a variety of reasons: structural uterine lesions (n = 103), HMB resolved on presentation (n = 85), on current hormonal therapy (n = 74), declined to participate (n = 47), anovulatory cycle pattern (n = 65), previous endometrial ablation (n = 29), wished future fertility desire (n = 15), medical disorders including current anticoagulation therapy (n = 11), and miscellaneous (n = 42). A total of 77 women with HMB consented to participate in the study (Fig 1). After a one month lead-in period, 67 women were randomly assigned to either MTP (n = 33: Naproxen^®^ n = 20, Nordette^®^, n = 13) or REA (n = 34). At the 12-month visit following initiation of treatment, 19/33 (58%) women in the MTP and 31/34 (91%) in the REA arm completed requisite questionnaires or visit and are included in our analysis.

Women had an average age of 42.3 ± 5.8 years, body mass index (BMI) of 28.9 ± 5.6 kg/m^2^, an average menstrual duration of 7.9 ± 3.2 days and a median PBLAC score of 300 (IQR, 218–479). With the exception of MMAS scores, baseline characteristics of women were similar (Table 1). Mean MMAS score was slightly higher in REA arm than MTP arm (46.6 ± 14.4 vs. 38.3 ± 18.1, p = 0.04). Similarly, cramps were more frequently reported in MTP arm (100% vs. 85%, p = 0.05).

**Table 1:** Baseline characteristics of the study population.

	Medical treatment arm (N = 33)	Surgical arm (N = 34)	P^†^
**Demographics and clinical characteristics**			
Age at consent date in years (mean ± SD)	42.8 ± 5.5	41.9 ± 6.0	0.51
BMI in kg/m^2^ (mean ± SD)	29.5 ± 5.9	28.4 ± 5.4	0.46
Gravidity (median [IQR])	3 (3–4)	3 (2–3)	0.27
Parity (median [IQR])	3 (2–3)	3 (2–3)	0.34
Number of previous Cesarean births (median [IQR])	0 (0–2)	0 (0–2)	0.53
Current contraception method, N (%)			0.68
*None*	1 (3.0)	4 (11.8)	
*Sterilization—Tubal Ligation*	14 (42.4)	14 (41.2)	
*Sterilization—Tubal Occlusion*	1 (3.0)	0 (0.0)	
*Sterilization—Partner Vasectomy*	14 (42.4)	13 (38.2)	
*Barrier methods*	3 (9.1)	3 (8.8)	
Personal/familial bleeding disorders, N (%)	0 (0.0)	1 (2.9)	0.99
Personal/family history venous thromboembolism, N (%)	5 (15.2)	4 (11.8)	0.73
History of fibroids, N (%)	5 (15.2)	4 (11.8)	0.73
History of hypertension, N (%)	2 (6.1)	4 (11.8)	0.67
History of diabetes, N (%)	2 (6.1)	2 (5.9)	0.99
History of tobacco use, N (%)	8 (24.2)	7 (20.6)	0.72
**Menstrual/bleeding characteristics**			
Average menstrual frequency in days (mean ± SD)	26.0 ± 3.7	26.6 ± 2.7	0.46
Average menstrual duration in days (mean ± SD)	8.1 (4.0)	7.7 (2.1)	0.60
Intermenstrual bleeding, N (%)	5 (15.2)	6 (17.6)	0.78
Post-coital spotting, N (%)	6 (18.2)	8 (23.5)	0.59
**Baseline work-up**			
Uterine sound length in cm (mean ± SD)	8.6 ± 0.9	8.7 ± 0.8	0.96
Last Pap screening result, N (%)			0.99
*Normal*	32 (97.0)	33 (97.1)	
Abnormal*	1 (3.0)	1 (2.9)	
Endometrial biopsy result, N (%)			0.24
*Normal*	32 (97.0)	30 (88.2)	
*Abnormal*	0 (0.0)	3 (8.8)**	
*Not Done*	1 (3.0)	1 (2.9)	
Pre-treatment hemoglobin in g/dL (median [IQR])	12.9 (12.3–13.6)	12.7 (11.5–13.7)	0.52
Serum Ferritin in ug/L (median [IQR])	15.5 (5.5–23.5)	10.5 (5.0–25.0)	0.73
Serum FSH in IU/L (median [IQR])	6.9 (4.4–8.6)	7.9 (5.2–11.0)	0.13
**Quantitative assessment of symptoms**			
PBLAC score (median [IQR])	290 (207–399)	312 (219–485)	0.36
Menorrhagia Quality of Life Survey Score “Menorrhagia Multi-Attribute Scale—MMAS” (mean ± SD)	38.3 ± 18.1	46.6 ± 14.4	0.04
Pain VAS (mean ± SD)	6 (5–7)	4 (2–6)	0.10
SF-12 physical scale (mean ± SD)	46.7 ± 8.6	49.1 ± 5.6	0.18
SF-12 mental scale (mean ± SD)	45.1 ± 10.0	45.6 ± 8.9	0.84

BMI, body mass index; IQR, interquartile range; PBLAC, pictorial blood loss assessment chart; SD, standard deviation; VAS, visual analog scale.

^†^ Chi-square or Fisher’s exact P value presented for categorical variables, t-test P value presented for age, body mass index, average menstrual frequency, average menstrual duration, uterine sound length, length of menstrual cycle, Menorrhagia Quality of Life Survey Score and SF-12 physical scale and SF-12 mental scale, Wilcoxon rank-sum P value presented for all remaining continuous or ordinal variables.

* Patients with abnormal Pap smear were diagnosed as: atypical squamous cells of undetermined significance (medical treatment arm) and abnormal; unsatisfactory for scanty cellularity (surgical arm).

** The three patients were diagnosed with chronic endometritis.

Twelve months after initial treatment, participants in the REA arm reported higher overall satisfaction than those in the MTP arm; 30 (96.8%) women in the REA arm described their experience as “totally satisfactory” in comparison to 12 (63.2%) women treated medically (p = 0.003). Quantitatively, MMAS scores were higher among women in the REA arm (p = 0.04 after adjustment for baseline measurements) (Table 2). Of the six components of the MMAS, participants’ response to “impact your bleeding currently has on work/daily routine” was significantly different between the two treatment arms; 29 (100%) women who responded to the MMAS in the REA arm reported “No interruptions to work/daily routine” versus 13 (72.2%) women among the 18 who responded to the MMAS in the MTP arm (p = 0.006) (S1 Appendix).

**Table 2:** Clinical outcomes in medical and surgical arms at 12 months of follow-up.

Characteristic	Medical treatment arm (N = 19)	Surgical arm (N = 31)	P^†^	Adjusted analysis P^‡^
PBLAC score (median [IQR])	15 (0–131)	0 (0–4)	0.003	0.003
Bleeding category, N (%)			0.15	
* Amenorrhea*	5 (26.3)	16 (51.6)		
* Irregular/Infrequent bleeding*	4 (21.1)	6 (19.4)		
* Prolonged bleeding*	2 (10.5)	-		
* Eumenorrhea*	8 (42.1)	9 (29.0)		
Menorrhagia Quality of Life Survey Score “Menorrhagia Multi-Attribute Scale—MMAS” (median [IQR])	100.0 (87.2–100.0)	100.0 (100.0–100.0)	0.12	0.04
Satisfaction with current treatment, N (%)			0.007	
* Totally satisfied*	12 (63.2)	30 (96.8)		
* Generally satisfied*	4 (21.1)	1 (3.2)		
* Acceptable improvement*	2 (10.5)	-		
* Unacceptable*	1 (5.3)	-		
SF-12 physical scale (mean ± SD)	54.2 ± 5.9	54.5 ± 4.2	0.82	0.99
SF-12 mental scale (mean ± SD)	49.8 ± 10.0	53.8 ± 6.6	0.11	0.06
Pain VAS (median [IQR])	0.4 (0.0–3.0)	0.0 (0.0–1.0)	0.08	0.16
Hemoglobin in g/dL (median [IQR])	13.2 (12.5–13.8)	13.4 (12.7–13.9)	0.38	0.19
Change in hemoglobin from baseline in g/dL (median [IQR])	0.0 (-0.6–0.7)	0.5 (0.0–2.2)	0.12	
Ferritin in ug/L (median [IQR])	25.0 (17.0–33.0)	26.5 (15.0–39.0)	0.60	0.24
Change in ferritin from baseline in ug/L (median [IQR])	4.0 (-1.0–16.0)	10.0 (4.0–22.0)	0.42	

IQR, interquartile range; PBLAC, pictorial blood loss assessment chart; SD, standard deviation; VAS, visual analog scale

^†^ T-test P value presented for SF-12 physical scale and SF-12 mental scale, Wilcoxon rank-sum P value presented for PBLAC score, Menorrhagia Quality of Life Survey Score MMAS, pain VAS, hemoglobin and ferritin levels, and satisfaction with current treatment, Chi-square or Fisher’s exact P value presented for remaining categorical variables.

^‡^ Adjusted analysis P-value from separate ANCOVA models adjusted for corresponding baseline measure. For PBLAC score, MMAS, pain VAS, and hemoglobin and ferritin levels the models were fit after applying a transformation (log(PBLAC+0.1), log(MMAS), log(pain VAS+0.1), log(hemoglobin), and log(ferritin)).

Furthermore, the median PBLAC score at 12 months was significantly lower among REA participants than those assigned to MTP (median [IQR], 0 [0–4] vs. 15 [0–131], p = 0.003). However, there were no differences in change from baseline to 12 months follow-up in hemoglobin (median [IQR], 0.0 [-0.6–0.7] vs. 0.5 [0.0–2.2] g/dL) or ferritin (median [IQR], 4 [-1.0–16.0] vs. 10.0 [4.0–22.0] ug/L) levels in MTP or REA arms, respectively. Lastly, REA participants had higher mean SF-12 mental scale scores than participants treated medically (53.8 ± 6.6 vs. 49.8 ± 10.0; p = 0.06 adjusted for baseline levels).

**Fig 2:**
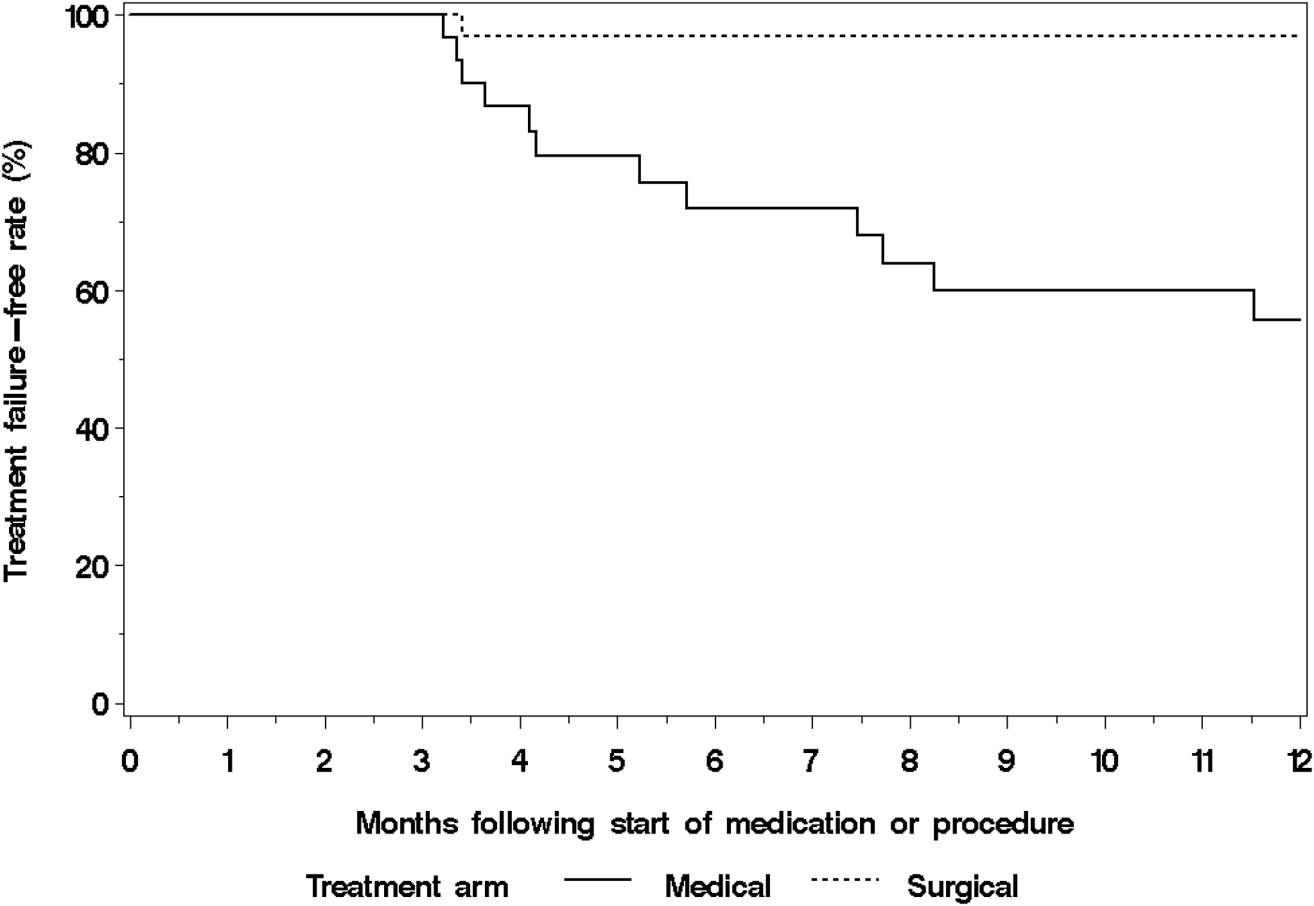
Kaplan-Meier curves for treatment failure (vaginal hysterectomy or endometrial ablation) by medical and surgical treatment arms.

**Fig 3:**
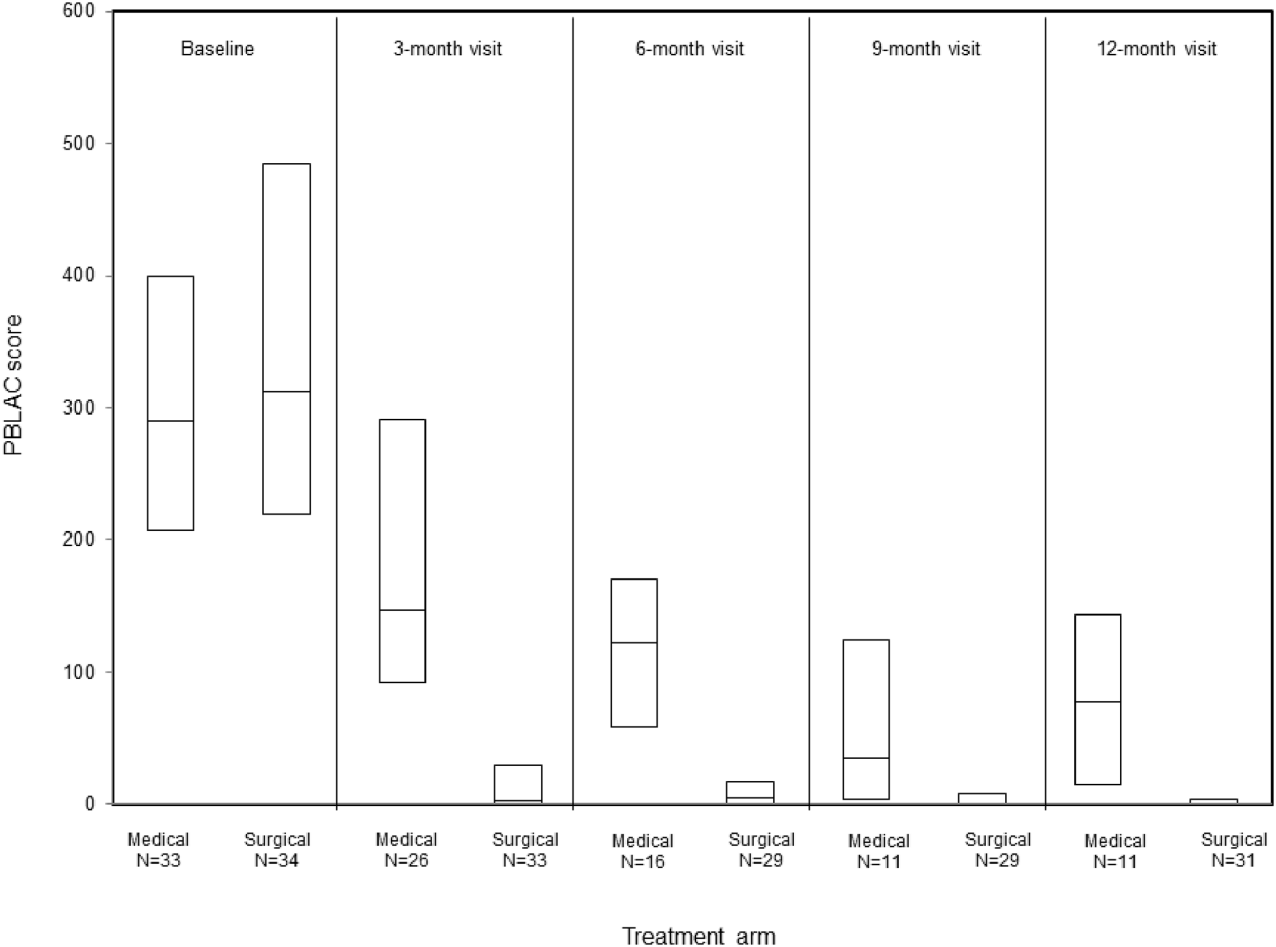
Pictorial blood loss assessment chart (PBLAC) score at follow-up among women in the medical and surgical arms. The top and bottom of each box denote the 25^th^ and 75^th^ percentiles and the middle line in each box denotes the median. In the surgical arm, the 25^th^ percentile at all of the follow-up visits was 0 and the median at the 9- and 12- month visits were also zero. Women who had an additional procedure were excluded from PBLAC score summary for subsequent visits.

Within 12 months following randomization, 13 women had an additional procedure. Four were lost to follow-up. Of the 9 women who participated in the 12-month visit, 8 were in the MTP arm and all were treated with REA. One woman in the REA arm was documented as treatment failure because she had LNG-IUS placed at 3 months for persistent HMB. Initial treatment to additional procedure interval was 0.47 ± 0.22 years. Thus, the treatment failure-free rate among women in REA arm was significantly higher than that of women in the MTP arm (log-rank p<0.001) (Fig 2). In a per-protocol analysis of the 12-month outcomes, women who had MTP alone (n = 11) had worse PBLAC scores, lower median MMAS, higher pain VAS, worse SF-12 mental scale and fewer proportion of women who reported total satisfaction compared to MTP who crossed over to REA (n = 8) or had REA only (n = 30) (Table 3). Finally, PBLAC score trend post randomization showed a dramatic and sustained reduction in REA arm in comparison to MTP only women (Fig 3).

**Table 3:** Per-protocol analysis of clinical outcomes in medical and surgical arms at 12 months of follow-up.

Characteristic	Medical treatment followed by endometrial ablation (N = 8)	Medical treatment arm (N = 11)	Surgical arm (N = 30)	P^†^	Adjusted analysis P^‡^
PBLAC score (median [IQR])	1 (0–3)	78 (15–143)	0 (0–4)	<0.001	<0.001
Bleeding category, N (%)				0.01	
* Amenorrhea*	4 (50.0)	1 (9.1)	15 (50.0)		
* Irregular/Infrequent bleeding*	2 (25.0)	2 (18.2)	6 (20.0)		
* Prolonged bleeding*	0 (0.0)	2 (18.2)	0 (0.0)		
* Eumenorrhea*	2 (25.0)	6 (54.5)	9 (30.0)		
Menorrhagia Quality of Life Survey Score “MMAS” (median [IQR])	100 (100–100)	88 (70–100)	100 (100–100)	0.001	<0.001
Satisfaction with current treatment, N (%)				<0.001	
* Totally satisfied*	8 (100.0)	4 (36.4)	29 (96.7)		
* Generally satisfied*	0 (0.0)	4 (36.4)	1 (3.3)		
* Acceptable improvement*	0 (0.0)	2 (18.2)	0 (0.0)		
* Unacceptable*	0 (0.0)	1 (9.1)	0 (0.0)		
SF-12 physical scale (mean ± SD)	56.4 ± 2.4	52.3 ± 7.2	55.1 ± 3.0	0.11	0.28
SF-12 mental scale (mean ± SD)	52.4 ± 8.9	47.8 ± 10.7	54.6 ± 4.8	0.01	0.004
Pain VAS (median [IQR])	0.1 (0.0–0.4)	2.6 (0.2–4.0)	0.0 (0.0–1.0)	0.01	0.01
Hemoglobin in g/dL (median [IQR])	12.9 (12.5–13.7)	13.3 (12.5–13.8)	13.3 (12.7–13.9)	0.55	0.22
Change in hemoglobin from baseline in g/dL (median [IQR])	-0.2 (-0.7–0.3)	0.0 (-0.6–1.4)	0.6 (0.0–2.2)	0.31	
Ferritin in ug/L (median [IQR])	25.5 (20.5–32.5)	25.0 (14.0–33.0)	26.0 (15.0–39.0)	0.57	0.22
Change in ferritin from baseline in ug/L (median [IQR])	9.5 (0.0–23.5)	4.0 (-1.0–16.0)	10.0 (4.0–22.0)	0.43	

IQR, interquartile range; MMAS, menorrhagia multi-attribute scale; PBLAC, pictorial blood loss assessment chart; SD, standard deviation; VAS, visual analog scale.

^†^ Comparing medical (N = 11) and surgical groups (N = 30). T-test P value presented for SF-12 physical scale and SF-12 mental scale, Wilcoxon rank-sum P value presented for PBLAC score, Menorrhagia Quality of Life Survey Score MMAS, pain VAS, menstrual diary scores, hemoglobin and ferritin levels, and satisfaction with current treatment, Chi-square or Fisher’s exact P value presented for remaining categorical variables.

^‡^ Comparing the medical (N = 11) and surgical (N = 30) groups) from separate ANCOVA models adjusted for corresponding baseline measure. For PBLAC score, MMAS, pain VAS, and hemoglobin and ferritin levels the models were fit after applying a transformation (log(PBLAC+0.1), log(MMAS), log(pain VAS+0.1), log(hemoglobin), and log(ferritin)).

In a post hoc analysis of the 17 “lost to follow-up” patients (MTP n = 14, REA n = 3), demographic and clinical characteristics were not different from those who completed surveys/visit at 12 months with the exception of parity (p = 0.02) (data not shown). Because the majority (n = 14; MTP [n = 11], REA [n = 3]) of these patients continued with their healthcare services at Mayo Clinic, we were able to determine if persistent or worsened HMB warranted any additional intervention following randomization. Within 12 months, 4/11 MTP patients had undergone REA for persistent HMB and 1 patient had levonorgestrel intrauterine systems inserted because of difficulty remembering taking medications; none of the 3 REA had further intervention. Finally, adverse effects were minor in both groups (Table 4).

**Table 4:** Number of adverse effects in the medical and surgical treatment within 12 months of treatment.

	Medical treatment arm*	Surgical arm
Headache		
* Mild headache*	2	
* Moderate headache*	4	
Gastrointestinal adverse events	2	
Depression	1	
Extremity edema	1	
Weight gain	1	
Increased blood pressure	1	
Urinary tract infection		1

*Some patients in the medical arm had more than 1 adverse event.

Table 5 shows the estimated direct and indirect costs by treatment group after 12 months of follow-up. All indirect cost analyses include costs for sanitary product use but report costs related to lack of activity and reduced work days were analyzed separately to reduce the likelihood of “double counting” costs related to limited capacity days. Analyses suggest that total direct medical costs of REA were higher than MTP at 12 months follow-up (mean, $5,331 vs. $2,901, mean difference and 95% CI $2,430 [$727, $4,852]). However, MTP was associated with significantly higher indirect costs regardless of how limited capacity days were valued. As a result there was no significant difference between total costs of MTP and REA when based solely on lack of activity or both limited capacity measures ($3,869 vs. $5,469, mean difference, 95% CI $1,600 [-$339, $4,089] both measures included). However, when only lost work days and cost of sanitary product use are considered in indirect cost analyses, total costs remained significantly higher for the REA group.

**Table 5:** Direct and indirect costs medical and surgical arms after 12 months of treatment.

	Medical treatment arm (N = 19)	Surgical arm (N = 31)	Surgical-medical mean difference (95% CI)
Direct medical costs	$2,901	$5,331	$2,430 ($727, $4,852)
* Direct medical costs (primarily hospital billed services)*^†^	$1,300	$3,494	$2,194 ($1,006, $3,978)
* Direct medical costs (primarily physician billed services)*	$1,601	$1,837	$236 (-$601, $1,087)
Indirect costs A*	$741	$124	-$617(-$1,225, -$135)
Indirect costs B*	$264	$27	-$237(-$465, -$54)
Indirect costs C*	$968	$138	-$830 (-$1,612, -$223)
Total Costs (direct costs + indirect costs A)	$3,642	$5,456	$1,814 (-$82, $4,304)
Total Costs (direct costs + indirect costs B)	$3,165	$5,358	$2,193 ($450, $4,614)
Total Costs (direct costs + indirect costs C)	$3,869	$5,469	$1,600 (-$339, $4,089)

All costs are described in mean values.

^†^ Primary hospital billed services as defined by Medicare billing practices.

* Indirect cost A refers to cost of sanitary products and lack of activity, indirect cost B refers to cost of sanitary products and reduced work days, and indirect cost C refers to cost of sanitary products, lack of activity, and reduced work days.

## Discussion

In this randomized controlled trial, we found radiofrequency endometrial ablation superior to oral contraceptive pills or Naprosyn therapy in the initial treatment of heavy menstrual bleeding, with lower menstrual blood loss, higher patient satisfaction rate, and better general and disease-specific quality of life at 12 months follow-up. Women initially allocated to MTP that subsequently crossed over to REA had similar improvements in clinical outcomes as those who received REA as initial therapy, whereas, women who received only MTP had worse outcomes. As expected, direct medical costs were higher for REA, and indirect costs were higher for MTP; however, average total costs of care were similar between treatment options when considering the impact of treatment on both activity levels and work capacity or on activity levels alone.

Prior studies compared OCPs or NSAID to placebo or other medications, medications versus hysterectomy, first generation endometrial ablation versus second generation, or compared various second generation devices to one another.[24–28] One study randomized women to first generation endometrial ablation (endometrial resection) or medical therapy that included combined oral contraceptive pills, oral progesterone, tranexamic acid or Danazol.[29] Similar to our findings, women allocated to endometrial resection were more likely to be totally satisfied and to find treatment acceptable; furthermore, although bleeding was reduced in the MTP arm, the reduction was less than that observed following endometrial ablation. A subsequent analysis of the 2-year follow-up data showed 59% of women allocated to medical therapy had undergone endometrial ablation or hysterectomy compared to 17% in the endometrial ablation arm.[30] At 5 years, 77% of patients in the MTP arm had undergone additional surgical therapy versus 27% in the endometrial ablation arm. [31]

Major strengths of our study include randomization design, use of objective clinical outcomes measures for heavy menstrual bleeding, and the inclusion of an economic analysis from the limited societal perspective. Despite numerous guidelines to include the impact of disease and treatment on productivity (indirect costs) and patient or family out of pocket costs, most cost analyses to date include only the direct medical costs or third party payments in analyses. [32, 33] We had the advantage of patient level data on resource use as well as prospectively assessed data on activity, work activities, and relevant sanitary product use to assess total costs of care. An analysis focused primarily on direct medical costs alone would have underestimated the impact of treatment we observed to have on the total disease clinical and economic burden.

As others have reported, recruiting patients for a randomized treatment trial of HMB is challenging [34] in part because patients often have a clear preference for a specific initial therapy and because of our strict adherence to study criteria. Thus, our findings will likely apply only to women with HMB who meet the selection criteria for our study. Similarly, our high lost to follow-up rate (42%) in the MTP arm is consistent with other studies and likely reflects the general dissatisfaction of patients allocated to medical therapy.[35] Indeed, our post hoc analysis showed 45% of MTP patients lost to follow-up had an additional intervention in contrast to none in the REA group. However, this limited our ability to quantify the incremental mean cost per quality-adjusted life gained for REA compared with MTP for a cost effectiveness analysis. In general, failure to reach stated sample size in randomized clinical trials would increase type II error (false negative or false equivalency in treatment arms) if the anticipated treatment arm differences used in the sample size calculations are consistent with the observed differences. However, because we recruited more than 50% of target accrual coupled with a lower than anticipated mean/median PBLAC score at 12 months in the surgical arm (anticipated 49 and observed 0) we believe the impact of small numbers is negligible on our primary outcome measures. Our inability to show differences in costs between treatment arms may reflect a type II error. Finally, the high crossover we observed made statistical analysis complicated, but the per-protocol analysis provided some evidence of the incremental benefit of REA. A future study may address the use of continuous administration of oral contraceptive pills in treating HMB.

## Conclusion

In conclusion, although medical therapy is beneficial to some women in treating HMB especially those who wish to retain fertility, the limited appeal, relatively lower patient satisfaction, and high discontinuation rates weaken the current recommendation for medical therapy as the first step in managing HMB. For women who meet criteria and have no future fertility desires, second generation endometrial ablation procedure may be offered as first line option as it demonstrates superior menstrual blood loss reduction, high patient satisfaction, and a near cost neutral alternative compared to medical therapy.

## Supporting information

S1 Appendix Baseline and 12-month visit responses to the Menorrhagia Multi-Attribute Scale (MMAS) per domain. (DOCX)

S2 Appendix CONSORT 2010 checklist. (DOC)

S3 Appendix Health economic checklist. (PDF)

S4 Appendix Study protocol. (PDF)
