# Supplementary material for: Medical therapy versus radiofrequency endometrial ablation in the initial treatment of heavy menstrual bleeding (iTOM Trial): A clinical and economic analysis
Source: PLoS One. 2017 Nov 15;12(11):e0188176. doi: 10.1371/journal.pone.0188176 (PMC5687740; doi:10.1371/journal.pone.0188176)
Supplement: S1 Appendix — (DOCX) [file pone.0188176.s001.docx]

**Appendix. Baseline and 12-month visit responses to the Menorrhagia Multi-Attribute Scale (MMAS) per domain**

|  | | **Medical (N=33)** | | | **Surgical (N=34)** | | | **P**^†^ | | |
| --- | --- | --- | --- | --- | --- | --- | --- | --- | --- | --- |
| **At baseline** | | | | | | | | | | |
| **Practical difficulties (Any practical difficulties you are currently  experiencing?)** | |  | | |  | | | 0.74 | | |
| No practical difficulties or extra precautions | | 0 (0.0) | | | 1 (2.9) | | |  | | |
| Carry extra sanitary protection, no other precautions | | 13 (39.4) | | | 14 (41.2) | | |  | | |
| Carry extra protection/clothes, risk of accident | | 10 (30.3) | | | 12 (35.3) | | |  | | |
| Severe problems w/ accidents, stay close to toilet | | 10 (30.3) | | | 7 (20.6) | | |  | | |
| **Social life (The impact your bleeding currently has on your  social life?)** | |  | | |  | | | 0.33 | | |
| Unaffected during cycle | | 3 (9.1) | | | 2 (5.9) | | |  | | |
| Slightly affected during cycle | | 14 (42.4) | | | 20 (58.8) | | |  | | |
| Limited during cycle, rarely make plans | | 16 (48.5) | | | 11 (32.4) | | |  | | |
| Devastated during cycle, no plans | | 0 (0.0) | | | 1 (2.9) | | |  | | |
| **Psychological health (Your current psychological health during your cycle?)** | |  | | |  | | | 0.14 | | |
| Have no worries, cope normally | | 2 (6.1) | | | 7 (20.6) | | |  | | |
| Experience some anxiety and worry | | 21 (63.6) | | | 22 (64.7) | | |  | | |
| Often feel down and worry about coping | | 8 (24.2) | | | 5 (14.7) | | |  | | |
| Feel depressed and cannot cope | | 2 (6.1) | | | 0 (0.0) | | |  | | |
| **Physical health and well-being (Your current physical health and well-being?)** | |  | | |  | | | 0.38 | | |
| Feel well and relaxed, not concerned | | 2 (6.1) | | | 1 (2.9) | | |  | | |
| Feel well most of the time, little concerned | | 4 (12.1) | | | 10 (29.4) | | |  | | |
| Often feel tired, do not feel especially well | | 23 (69.7) | | | 20 (58.8) | | |  | | |
| Feel very tired, do not feel well at all | | 4 (12.1) | | | 3 (8.8) | | |  | | |
| **Work/daily routine (Impact your bleeding currently has on work/daily  routine?)** | |  | | |  | | | 0.70 | | |
| No interruptions to work/daily routine | | 1 (3.0) | | | 2 (5.9) | | |  | | |
| Occasional disruptions to work/daily routine | | 8 (24.2) | | | 11 (32.4) | | |  | | |
| Frequent disruptions to work/daily routine | | 20 (60.6) | | | 19 (55.9) | | |  | | |
| Severe disruptions to work/daily routine | | 4 (12.1) | | | 2 (5.9) | | |  | | |
| **Family life/relationships (Impact your bleeding currently has on family/ relationships?)** | |  | | |  | | | 0.02 | | |
| Unaffected during my cycle | | 2 (6.1) | | | 2 (5.9) | | |  | | |
| Suffer some strain during my cycle | | 14 (42.4) | | | 25 (73.5) | | |  | | |
| Suffer quite a lot during my cycle | | 16 (48.5) | | | 6 (17.6) | | |  | | |
| Severely disrupted as a result of my cycle | | 1 (3.0) | | | 1 (2.9) | | |  | | |
| **At 12-month visit (intention to treat analysis)** | | | | | | | | | | |
|  | | **Medical (N=19)** | | | **Surgical (N=31)** | | | **P** | | |
| **Practical difficulties (Any practical difficulties you are currently  experiencing?)** | |  | | |  | | | 0.18 | | |
| No practical difficulties or extra precautions | | 14/18 (77.8) | | | 27/29 (93.1) | | |  | | |
| Carry extra sanitary protection, no other precautions | | 4/18 (22.2) | | | 2/29 (6.9) | | |  | | |
| **Social life (The impact your bleeding currently has on your  social life?)** | |  | | |  | | | 0.05 | | |
| Unaffected during cycle | | 15/18 (83.3) | | | 29/29 (100.0) | | |  | | |
| Slightly affected during cycle | | 3/18 (16.7) | | | 0/29 (0.0) | | |  | | |
| **Psychological health (Your current psychological health during your cycle?)** | |  | | |  | | | 0.23 | | |
| Have no worries, cope normally | | 13/18 (72.2) | | | 26/29 (89.7) | | |  | | |
| Experience some anxiety and worry | | 5/18 (27.8) | | | 3/29 (10.3) | | |  | | |
| **Physical health and well-being (Your current physical health and well-being?)** | |  | | |  | | | 0.07 | | |
| Feel well and relaxed, not concerned | | 12/18 (66.7) | | | 26/29 (89.7) | | |  | | |
| Feel well most of the time, little concerned | | 5/18 (27.8) | | | 3/29 (10.3) | | |  | | |
| Often feel tired, do not feel especially well | | 1/18 (5.6) | | | 0/29 (0.0) | | |  | | |
| **Work/daily routine (Impact your bleeding currently has on work/daily  routine?)** | |  | | |  | | | 0.006 | | |
| No interruptions to work/daily routine | | 13/18 (72.2) | | | 29/29 (100.0) | | |  | | |
| Occasional disruptions to work/daily routine | | 4/18 (22.2) | | | 0/29 (0.0) | | |  | | |
| Frequent disruptions to work/daily routine | | 1/18 (5.6) | | | 0/29 (0.0) | | |  | | |
| **Family life/relationships (Impact your bleeding currently has on family/ relationships?)** | |  | | |  | | | 0.24 | | |
| Unaffected during cycle | | 15/18 (83.3) | | | 28/29 (96.6) | | |  | | |
| Slightly some strain during my cycle | | 2/18 (11.1) | | | 1/29 (3.4) | | |  | | |
| Suffer quite a lot during my cycle | | 1/18 (5.6) | | | 0/29 (0.0) | | |  | | |
| **At 12-month visit (per protocol analysis)** | | | | | | | | | | |
|  | **Medical treatment followed by ablation**  **(n=8)** | | **Medical**  **(n=11)** | | | **Surgical**  **(n=30)** | | **P** | | |
| **Practical difficulties (Any practical difficulties you are currently  experiencing?)** | |  | | | |  | | 0.03 | | |
| No practical difficulties or extra precautions | | 8 (100.0) | | 6/10 (60.0) | | | 26/28 (92.9) | |  |  |
| Carry extra sanitary protection, no other precautions | | 0 (0.0) | | 4/10 (40.0) | | | 2/28 (7.1) | |  |  |
| **Social life (The impact your bleeding currently has on your  social life?)** | |  | |  | | |  | | 0.01 |  |
| Unaffected during cycle | | 8 (100.0) | | 7/10 (70.0) | | | 28/28 (100.0) | |  |  |
| Slightly affected during cycle | | 0 (0.0) | | 3/10 (30.0) | | | 0/28 (0.0) | |  |  |
| **Psychological health (Your current psychological health during your cycle?)** | |  | |  | | |  | | 0.01 |  |
| Have no worries, cope normally | | 8 (100.0) | | 5/10 (50.0) | | | 25/28 (89.3) | |  |  |
| Experience some anxiety and worry | | 0 (0.0) | | 5/10 (50.0) | | | 3/28 (10.7) | |  |  |
| **Physical health and well-being (Your current physical health and well-being?)** | |  | |  | | |  | | 0.003 |  |
| Feel well and relaxed, not concerned | | 8 (100.0) | | 4/10 (40.0) | | | 25/28 (89.3) | |  |  |
| Feel well most of the time, little concerned | | 0 (0.0) | | 5/10 (50.0) | | | 3/28 (10.7) | |  |  |
| Often feel tired, do not feel especially well | | 0 (0.0) | | 1/10 (10.0) | | | 0/28 (0.0) | |  |  |
| **Work/daily routine (Impact your bleeding currently has on work/daily  routine?)** | |  | |  | | |  | | <0.001 |  |
| No interruptions to work/daily routine | | 8 (100.0) | | 5/10 (50.0) | | | 28/28 (100.0) | |  |  |
| Occasional disruptions to work/daily routine | | 0 (0.0) | | 4/10 (40.0) | | | 0/28 (0.0) | |  |  |
| Frequent disruptions to work/daily routine | | 0 (0.0) | | 1/10 (10.0) | | | 0/28 (0.0) | |  |  |
| **Family life/relationships (Impact your bleeding currently has on family/ relationships?)** | |  | |  | | |  | | 0.03 |  |
| Unaffected during cycle | | 8 (100.0) | | 7/10 (70.0) | | | 28/28 (100.0) | |  |  |
| Slightly some strain during my cycle | | 0 (0.0) | | 2/10 (20.0) | | | 0/28 (0.0) | |  |  |
| Suffer quite a lot during my cycle | | 0 (0.0) | | 1/10 (10.0) | | | 0/28 (0.0) | |  |  |

^†^Chi-square or Fisher’s exact P value
