# Supplementary material for: Medical therapy versus radiofrequency endometrial ablation in the initial treatment of heavy menstrual bleeding (iTOM Trial): A clinical and economic analysis
Source: PLoS One. 2017 Nov 15;12(11):e0188176. doi: 10.1371/journal.pone.0188176 (PMC5687740; doi:10.1371/journal.pone.0188176)
Supplement: S3 Appendix — (PDF) [file pone.0188176.s003.pdf]

Special Instructions For Authors Of Health Economics Manuscripts*

In addition to the general instructions for authors and other guidelines applicable to their study (for example, CONSORT guidelines for a randomized, controlled trial), authors of health economics manuscripts should consider the following issues specific to such studies and address them in the manuscript and/or submission letter. This checklist should be included with the general manuscript checklist.

Not applicable or issue addressed in:		
Manuscript Page Number	Submission Letter	Issue to be addressed
**Title**		
	✓	Includes the type of economic evaluation and any comparisons if applicable
**Introduction**		
5		Includes the type of economic evaluation and the comparative interventions in the aim of the study
**Materials and Methods**		
8		Includes the perspective (point of view) and time frame of the economic evaluation
8		Adequately describe alternative strategies or interventions
Not Relevant		Include a "do nothing" strategy or a rational for its exclusion
9		Primary outcome analysis is well defined and established *a priori* or the rational for a *post hoc* analysis is defined
Not Relevant		Include and justify the details and parameters of the model or decision analysis tree
8		Include consideration of long-term costs and benefits
Not Relevant		Document the reliability of information sources
Not Relevant		Sensitivity analyses have reasonable ranges and are based on evidence
8		Describe methods for estimating quantities and unit costs
Not Relevant		Include currency conversion and discount rates or rational for excluding these
Not Relevant		Adjust for timing both of costs and of benefits or include a rational for excluding this
**Results**		
25 (Table 5)		Present data both in aggregated and disaggregated forms
Not Relevant		Cost-benefit analyses specify the type of evaluation (that is benefits-to-cost ratio, net present value, or net benefits as percentage of costs)
Not Relevant		Express results of any break-even equation in monetary terms
8		Specify if human capital or willingness to pay approach is used in cost-benefit analyses
Not Relevant		Express cost-effectiveness analyses data as cost per unit of effectiveness (cost-effectiveness ratio)
Not Relevant		Express treatment cost-effectiveness comparisons as incremental cost-effectiveness ratios
Not Relevant		Express cost-utility analysis results as cost per quality-adjusted life-year

**Definitions for types of economic evaluations**	
Cost-benefit analysis	Compares monetary cost and benefits of alternative strategies
Cost-effective analysis	Compares the cost of alternative strategies that have different clinical outcomes
Cost-minimization analysis	Compares the costs of alternative strategies that have clinically equivalent outcomes
Cost-utility analysis	Compares the costs of alternative strategies using quality of life outcome measures

*Adapted from Vintzileos and Beazoglou. Am J Obstet Gynecol 2004;191:1070-6.
