# Supplementary material for: Medical therapy versus radiofrequency endometrial ablation in the initial treatment of heavy menstrual bleeding (iTOM Trial): A clinical and economic analysis
Source: PLoS One. 2017 Nov 15;12(11):e0188176. doi: 10.1371/journal.pone.0188176 (PMC5687740; doi:10.1371/journal.pone.0188176)
Supplement: S4 Appendix — (PDF) [file pone.0188176.s004.pdf]

Prospective Randomized Trial of Medical Therapy versus Radiofrequency Endometrial Ablation in the Initial Treatment of Menorrhagia: Treatment Outcomes and Economic Analysis

Principal Investigator: Abimbola Famuyide, MBBS.
Minimally Invasive Gynecological Surgery
Mayo Clinic, Rochester

Confidential and Proprietary Information of Mayo Clinic, Rochester, MN

Outline of Proposal

1.0 Abstract
2.0 Specific Aims
    Specific Aim #1
    Specific Aim #2
3.0 Hypotheses
    Study Hypothesis for Specific Aim #1
    Study Hypothesis for Specific Aim #2
4.0 Background and Significance
5.0 Experimental Design and Methods
    - Overview
    - Study Population
        - Patient Selection
        - Inclusion Criteria
        - Exclusion Criteria
    - Measure of Study Variables
        - Patient Flow
        - Outcome Measurements
    - Statistical Analysis
        - Power Calculation
        - Statistical Analysis for the Efficacy and Safety Endpoints (specific aim #1)
        - Outcome Measurements of Cost Analysis (specific aim #2)
6.0 Adverse Events
        - Serious Adverse Events
        - Unanticipated Adverse Device Effects
        - Anticipated Effects of Endometrial Ablation
        - Anticipated Effects of Non-Steroidal Anti-Inflammatory Drugs (Naproxen Sodium)
        - Anticipated Effects of Oral Contraceptive Pills (OCP)
        - Reporting of Adverse Events
        - Data and Safety Monitoring Plan
7.0 Ethical Considerations (Human Studies)
8.0 Study Expectations and Limitations
9.0 Budget
10.0 Consent Form
11.0 Literature Cited
12.0 Observations
13.0 Appendices

1.0 Abstract

Excessive menstrual loss (menorrhagia) is a common condition that affects women of reproductive age, and can result in anemia, chronic fatigue and lost wages from work. The traditional first line management involves treatment with oral contraceptives or non-steroidal anti-inflammatory agents, both of which are often ineffective with significant unpleasant side-effects. Many women ultimately undergo hysterectomy, a major operative procedure associated with increased costs, loss of feeling of womanhood, debilitating complications and on rare occasions, death. The newer global endometrial ablation devices allow the destruction of the endometrial lining without the removal of the uterus in an ambulatory surgery setting. They offer a safe and effective alternative to hysterectomy with minimal risks and unpleasant side-effects. Presently, global endometrial ablation is offered as an alternative to hysterectomy, after medical intervention has failed. This study will determine the role of global endometrial ablation in the initial management of menorrhagia. Women seeking treatment for menorrhagia will be offered randomization to medical treatment arm with oral contraceptive pills or global endometrial ablation arm. This study will be the first to compare clinical efficacy and costs between oral contraceptive pills and global endometrial ablation in the initial management of menorrhagia. The results of this study could potentially change the management of menorrhagia and impact millions of women who suffer from this condition in the US and world-wide.

2.0 Specific Aims:

2.1 Specific aim #1: To determine if global endometrial ablation (GEA) is more effective than medical therapy in the initial management of menorrhagia.

In order to evaluate specific aim #1, both treatment arms will be compared using the following outcome measures at 6 and 12 months:

1. Menstrual blood loss (MBL) as measured by pictorial blood loss assessment chart (PBLAC).
2. Patients' satisfaction rates and health related Quality of Life (using SF-12 and menorrhagia specific questionnaire).

3. Time to failure of treatment (failure is defined as hysterectomy or ablation using a Cox proportional hazard model).
4. Hemoglobin and Ferritin levels pre and post-treatment
5. Severity of dysmenorrhea symptoms (using Dysmenorrhea Score) and Premenstrual Syndrome (using PMS-Diary)

2.2 Specific aim #2: To determine disease-specific resource utilization and costs associated with the treatment alternatives and the cost effectiveness of global endometrial ablation (GEA) compared with medical treatment (oral contraceptive pills) in the initial management of menorrhagia.

1. The endpoint for this specific aim is the assessment of total disease-specific costs associated with the two treatment groups at 12 months. Costs assessed will include direct medical costs of care, patient costs (sanitary product use), as well as indirect costs (lost workdays).
2. A cost effectiveness analysis will be conducted assessing the incremental mean costs per quality-adjusted life year gained with GEA compared with medical treatment should effectiveness of treatment be established with observed gains in health-related quality of life (SF-12®).

2.3 Specific aim #3: To compare the efficacy of radiofrequency ablation in women who had dilatation and curettage for endometrial thinning compared to those who did not. (The same endpoints of aim#1 will be used for this specific aim)

3.0 Study Hypotheses:

3.1 Study Hypotheses for Specific aim #1: "Global endometrial ablation is a safe and effective alternative to medical therapy in the initial treatment of menorrhagia in women who have no future fertility desire." That is, comparisons between the two treatment groups on measures for Specific Aims 2.1.1- 2.1.5 will show statistically significant differences in favor of the GEA group of patients.

3.2 Study Hypotheses for Specific aim#2: "Total costs associated with global endometrial ablation as an initial management of menorrhagia will be similar or higher than those for patients treated with medical therapy and the quality-adjusted life years gained are obtained at reasonable cost."

3.3 Study Hypotheses for Specific aim#3:"Surgical endometrial thinning will improve the efficacy of global endometrial ablation."

4.0 Background and Significance

Menorrhagia accounts for approximately 20% of office visits by women of reproductive age in the US and worldwide. (1,2) As a result of this condition, many women miss work, and are socially incapacitated with resultant emotional and psychological stress. It has been estimated that approximately $2 billion is spent annually in the US on the treatment of menorrhagia. (3,4,5)

The American College of Obstetricians and Gynecologists, in its practice guidelines, recommend oral contraceptive pill therapy for the initial management of menorrhagia. (6). Medical therapy typically consists of oral contraceptive therapy (OCP), cyclical progesterone or non-steroidal inflammatory agents (NSAID) if OCP is contraindicated. Response to OCP therapy is inconsistent and only 40% report satisfaction with such treatment. As many as 76% will experience unpleasant side effects such as headaches, bloating or breast tenderness. Although cyclical progestogens may be useful in irregular vaginal bleeding, they are ineffective in treating women with menorrhagia. NSAIDs are frequently prescribed in clinical practice to reduce menstrual blood flow and alleviate dysmenorrhea. However, gastro-intestinal complaints are common especially when administered over a prolonged period of time. There are differences in the clinical efficacy of the various NSAIDs, with some women responding well to one agent and poorly to the other. Mefenamic acid and naproxen sodium are the most frequently used NSAIDs in studies with comparable efficacy, but naproxen sodium is considerably cheaper, and may cause more gastrointestinal side effects.

There is no difference in efficacy between NSAIDs and OCP therapy in the treatment of menorrhagia (7-15)

Hysterectomy has been the mainstay of treatment following failed medical therapy because of its effectiveness and high patient satisfaction rates. However, it is expensive, morbidity rates approach 40% and 6-10/10,000 patients die as a direct complication of the surgical procedure. Moreover, the uterus is histopathologically normal in half of the 200,000 hysterectomies performed annually in the US for menorrhagia. Many therefore, question the need for the removal of an anatomically normal uterus. Additionally, some women associate hysterectomy with the loss of womanhood and sexual dysfunction despite evidence to the contrary.

Recent advances in global endometrial ablation (GEA) devices allow for a safe ablation of the endometrium without the need for expertise in hysteroscopic surgery. Energy (radiofrequency, thermal, microwave) is delivered directly to the endometrium causing necrosis and eventual scaring, leading to hypomenorrhea or even amenorrhea. Presently, five devices are FDA approved for use in the US. Regardless of the device type or energy utilized, 85-95% of patients are satisfied and 15-40% report amenorrhea following treatment. At our institution, radiofrequency ablation (NovaSure™) is employed because of its shorter duration of treatment (less than two minutes) thus allowing out-patient treatment under conscious sedation, rapid recovery and early return to work, typically within 24 hours post-treatment. Additionally, patients experience less post-operative pain (3%) and superior amenorrhea rates (40-59%) at 1 year post-treatment compared to other devices. Five-year data also suggest that GEAs reduce the need for hysterectomy. (16-18)

Currently, GEA is recommended only after the initial medical therapy has failed. This recommendation is based on the assumption that medical therapy is effective, more tolerable and cheaper in comparison to surgical therapy. Whilst these assumptions may be valid in relation to hysterectomy and older hysteroscopic endometrial resections, it may not be true of the newer global endometrial ablation techniques since they are less invasive with very high acceptance rates and fewer adverse effects

and may therefore have a role in the initial management of menorrhagia. Presently, there are no published efficacy or trial-based economic analyses comparing medical therapy with the newer global endometrial ablation procedures.

5.0 Experimental Design and Methods

5.1 Overview:

This is a single center non-blinded, randomized, controlled clinical trial. Women of reproductive age group without future fertility desire, seeking first time treatment for menorrhagia will be asked to participate in the trial. Subjects who agree will be entered into a one month lead-in period, during which PBLAC, dysmenorrhea and premenstrual diaries for a menstrual cycle will be kept. Enrolled subjects will be randomized to either medical therapy with 30 microgram monophasic oral contraceptive pills (naproxen sodium if OCP therapy is contra-indicated) or radiofrequency endometrial ablation using the NovaSure® device. Half the endometrial ablation subjects will be randomly assigned to undergo endometrial curettage immediately preceding the ablation procedure. Although endometrial pre-treatment is not required for a successful outcome, we believe that prior endometrial curettage performed to thin the endometrium may increase amenorrhea rates (unpublished data). At our institution in 2005, 511 women underwent surgical treatment for menorrhagia; 308 of these were endometrial ablations. It is anticipated that a total of 240 patients will be recruited over a 12 month period and randomized 1:1 to each arm

5.2 Study population

5.2.1 Patient selection

Subjects will be enrolled if they satisfy the inclusion and exclusion criteria, and agree to be randomized to medical therapy or radiofrequency endometrial ablation. Information on all subjects approached will be recorded in a screening log.

Subjects that meet the inclusion criteria and agree to evaluation as scheduled in the study protocol will be required to sign a written consent prior to enrollment.

5.2.2 Inclusion criteria

1. Adult female, ages 30-55, who is pre-menopausal and for whom childbearing is complete
2. Subjective symptom of excessive menstrual loss
3. Normal uterine cavity length (≥ 4cm) with a sound measurement of ≤10cm documented by sonohystogram or hysteroscopy in the preceding 6 months
4. At least one normal Pap Test within past 3 years, and no unexplained abnormal Pap Tests within 6 months of procedure
5. Prior history of permanent sterilization or use of reliable non-hormonal contraception during the 14 month study period or history of vasectomy in partner
6. Freely agree to participate in the study including all study related procedures and evaluations, and document this agreement by signing the informed consent document

5.2.3 Exclusion criteria

1. Pregnancy or desire for future childbearing
2. Active lower genital infection at the time of procedure
3. Active urinary tract infection at the time of procedure
4. Active pelvic inflammatory disease (PID) or recurrent chronic PID
5. Endometrial neoplasia, determined by endometrial biopsy taken within 12 months of study entry
6. Current or past history of cervical or endometrial cancer
7. Uterine sound measurement greater than 10cm
8. Submucous leiomyoma greater than 2cm or cavity distorting leiomyoma
9. History of myomectomy or classical cesarean section
10. Previous endometrial ablation

11. Oral hormonal treatment in the preceding 3 months, hormone releasing intrauterine contraceptive in the preceding month, or injectable hormone treatment in the preceding 12 months
12. Contraindication to hormonal therapy <u>and</u> non-steroidal anti-inflammatory agents (appendix-).
13. History of a coagulopathy or endocrinopathy
14. Inability to follow up at 12 months

5.3 Measurements of Study Variables:

5.3.1 Patient flow:

a) Baseline and Enrollment:

Subjects who meet the eligibility criteria will be required to sign an informed consent form and HIPAA authorization prior to entering into the one-month lead-in period. Subjects will receive identification numbers at this time and will be considered enrolled into the study. During the pre-enrollment period, baseline information on a menstrual cycle will be collected using PBLAC, dysmenorrhea and premenstrual symptoms diaries. All subjects will undergo a history and physical examination including pelvic examination. A Pap test using ThinPrep® will be obtained if none has been taken in the preceding 6 months. Office hysteroscopy will be performed to exclude cavity distorting submucous leiomyoma. Pipelle endometrial biopsy will be obtained to exclude endometrial hyperplasia or cancer. Uterine sound measurement will be read off the Pipelle catheter and recorded. At enrollment, blood will be drawn for baseline hemoglobin, serum ferritin, and serum HCG if pregnancy is suspected. To control for post treatment amenorrhea due to menopause, follicular stimulating hormone (FSH) levels will be assayed (and repeated at 12 months in the ablation arm). Subjects will be requested to fill out quality of life questionnaires.

b) Randomization:

Enrolled patients will be randomized to medical therapy or radiofrequency endometrial ablation. Randomization will be done with stratification based on: age (< 45 years, ≥ 45 years), parity (<2, ≥ 2) and body mass index (BMI< 30, BMI ≥ 30). The randomization will be performed using a dynamic allocation approach based on the Pocock-Simon method to ensure a balance in the treatment groups across the stratification levels. The randomization assignment for each patient will be obtained upon entering the patient's stratification levels into a web-based application created and supported by the Division of Biomedical Statistics and Informatics.

c) Medical therapy arm:

After the one-month lead-in period, subjects will be prescribed 4 packets of 30-microgram estradiol/150mcg levonorgestrel monophasic oral contraceptive pills (Nordette®). Using this formulation, (Frazer, 1991) showed a 43% reduction in menstrual blood flow in women with menorrhagia. (8)

Each packet consists of a fixed dose of 30 microgram estradiol and a 150 mg dose of levonorgestrel for the first 21 days of the cycle, followed by a 7 day pill free period. Subjects will be instructed to administer the pills orally, starting 5 days after the start of menstrual blood flow, continuing cyclically, thus allowing for withdrawal bleeding after the 21 day pill cycle. Subjects are expected to continue treatment for 12 consecutive months (study duration) and follow-up at 3, 6, 9, and 12 months for further evaluation and to receive additional prescriptions as needed.

Subjects who are unable to tolerate OCP or are unwilling to administer OCP will be prescribed naproxen sodium pills. The latter will be administered as follows; 500mg with onset of menses, then 250mg three times daily for the duration of the menses (or maximum of five days). Each subject will receive 48 pills (16 per

menstrual cycle), renewable every 3 months. Compliance to treatment will be evaluated at each follow up visit by checking for the medication. If a subject fails to comply with the protocol requirements, her data will be censored and all data collected prior to loss of compliance will be analyzed.

Patients randomized to the medical therapy arm will remain on either OCP or naproxen sodium pills for the duration of the study, 12 consecutive months following enrollment. In the event of intolerable side effects, in which a patient requests to discontinue medication or the treating physician discontinues the randomized form of treatment, the patient will be considered a failure for this treatment arm. However for the economic analysis all women will be followed for 12 months to assess disease-specific resource utilization and costs.

d) Radiofrequency endometrial ablation (NovaSure®) arm:

Subjects will undergo radiofrequency endometrial ablation within 4 weeks of randomization. The procedure will be performed per the NovaSure® Instructions for Use (IFU) by 1 of 4 physicians (Dr. Famuyide, Dr. Hopkins, Dr. Laughlin, or Dr. Breitkopf) and will occur at any time during the menstrual cycle, without endometrial pre-treatment. All subjects will receive intravenous Fentanyl, Propofol and Midazolam as determined by the attending anesthesiologist sufficient to induce conscious sedation. The cervix will be dilated to 8mm, in order to accommodate the 7.2mm endometrial ablation probe. The uterine cavity length and width will be key-entered into the radiofrequency generator. The treatment time is dependent on thickness of the endometrial lining and typically averages 90 seconds, but no longer than 120 seconds. The depth of tissue destruction is directly correlated to the measured tissue impedance, pre-set at 50 Ohms which translates to a depth of 5-7mm in the myometrium. Both

the procedure time (device insertion to device removal), and the treatment time (time to deliver energy) will be recorded. Subjects will be sent to the out-patient floor to recover from surgery. The time spent in the recovery room will be recorded. Endometrial thinning will be carried out using suction curettage in 50% of the cases included in the ablation group. Random assignment for this treatment will be included in the overall randomization plan. Subjects will be dismissed home once standard out-patient surgery dismissal criteria are met. Follow-up will be arranged 3 months after surgery. Patients will be instructed to contact the PI (Dr. Famuyide) sooner if needed. All peri-operative and intra-operative adverse events and data will be recorded (see appendix).

Complications associated with the NovaSure® device and procedure will be recorded on the case report forms. In the event of technical failure, when radiofrequency treatment has not been initiated, the procedure will be aborted. The subject will remain enrolled in the study and a second attempt will be performed per institutional standards. If the NovaSure® procedure has been completed, (as indicated by illumination of the procedure completed light), then this patient will not be re-ablated.

In the event of a failed Cavity Integrity Assessment (CIA), with evidence of and/or documentation of a uterine perforation, the procedure will be aborted and the event documented on the applicable case report forms. The subject will be allowed to recover and will return to the treating facility 6 weeks post procedure, to re-attempt the ablation procedure.

e) Post-treatment follow-up:

Three visits and a phone contact are planned. Subjects will be instructed to contact the PI (Dr. Famuyide) if the need arises between the scheduled visits.

3, 6 and 12 month follow up visit- Data will be collated for menstrual cycles 1-3, 4-6, 10-12; bleeding score (PBLAC), dysmenorrhea (visual analogue scale), side effects profile (irregular vaginal bleeding, vaginal discharge, bloating,

headache, mastalgia, mood swing, dyspepsia, heartburn, hematemesis or melena), SF-12®, satisfaction rates and the number of days absent from work as a result of bleeding or adverse effect of treatment. The incidence of additional treatment (hysterectomy or ablation) will be recorded. Subjects in the medical therapy arm will have a pill count performed. Blood will be drawn for hemoglobin, Ferritin in all patients at 3 and 12 months. For patients in the ablation arm including crossovers from medical arm, FSH levels will be assayed at 12months post treatment. Pelvic examination will be performed when clinically indicated.

9 month phone contact- Subjects will be contacted by phone to inquire about problems that need further evaluation. Appointments will be scheduled if needed.

The incidence of additional treatment (hysterectomy or ablation) will be recorded. Subjects will be required to mail in diaries and questionnaires as detailed in the 3 month visit.

5.3.2 Outcome measurements:

End points:

A) Efficacy of treatment end points <u>(specific aim#1):</u>

1. Bleeding score (PBLAC) <u>(primary endpoint)</u>
2. Bleeding patterns including rate of amenorrhea/ eumenorrhea.
3. Menorrhagia specific questionnaire
4. Satisfaction
5. Quality of life questionnaire.
6. Time to event (hysterectomy or ablation)
7. Change in Hemoglobin
8. Change in Ferritin

9. Dysmenorrhea (visual analogue scale)
10. Premenstrual symptoms (PMS diary)
10. Adverse effects of treatment

B) Economic endpoints <u>(specific aim #2):</u>

1. Disease-specific direct medical costs (<u>primary endpoint</u>)
2. Patient and indirect costs (sanitary product use and lost workdays)
3. Total costs (direct medical costs, patient costs, as well as indirect costs associated with lost workdays or limited abilities).
4. Incremental mean cost per quality-adjusted life year gained for ablation compared with medical therapy using a preference based measure of health derived from the SF-12® (Brazier and Roberts, 2004; Brazier, Roberts, Deverill, 2002) (19,20).

5.3.2.1 Outcome Measurements (specific aim #1):

1- Bleeding Score (PBLAC) [specific aim #1 (primary endpoint)]: Subjects will be asked to complete the PBLAC at baseline and at 3, 6, 9 and 12 months after the intervention. Score of the PBLAC will be used as a surrogate (semi-quantitative) measure of menstrual blood loss in women included in the study (21).

2- Evaluation of the bleeding pattern: During the follow up visits/call, the menstruation pattern of the participating women will be evaluated. A *<u>bleeding episode</u>* will be defined as any set of one or more bleeding days (either consecutive or separated by only one bleeding-free days) bounded at each end by two or more bleeding-free days. The bleeding pattern will be analyzed using a 90 day reference period and divided into six groups (based on WHO classification of clinically important bleeding patterns (22,23):

1. Amenorrhea (no bleeding during the reference period),
2. Infrequent bleeding (fewer than 3 bleeding episodes),
3. Frequent bleeding (more than 5 bleeding episodes),
4. Irregular bleeding (between 3 and 5 episodes with less than 3 bleeding-free intervals of length 14 days or more),

5. Prolonged bleeding (1 or more bleeding episodes lasting 14 days or more)
6. Eumenorrhea “Normal pattern" (none of the above patterns).

3- Time to event (hysterectomy, or ablation): The time to event will be estimated from randomization until the occurrence of the first event/procedure.

4- Menorrhagia specific questionnaire

5- Satisfaction Rates of study subjects will be ascertained for treatment outcomes by asking patients to choose one of the following 5 categories: totally satisfied, generally satisfied with treatment, cure, acceptable improvement in symptoms, or treatment unacceptable. Additionally, subjects will be asked to indicate if they would be willing to undergo the same treatment again or would recommend the treatment to family or friends.

6- Health-related quality of life will be measured using the SF-12® measure of health for all subjects. Measurements will be made before any treatment intervention (baseline) and 6 and 12 months post-treatment. (See attached pdf copy of the SF-12 (version1) survey instrument).

7- Dysmenorrhea Pain Scale: A simple visual analogue scale (0-10) will be used to evaluate pain associated with menstrual blood loss evaluation by PBLAC at 3, 6, 12 months follow up visits.

8- Premenstrual Symptoms Diary: Subjects will be asked to document the presence of any of the following symptoms preceding a menstrual cycle, at 3, 6, 12 months visit; bloating, breast discomfort, irritability, anger, moodiness, headaches, fluid retention.

9- Serum hemoglobin and ferritin at 3 and 12 months, FSH levels at 12 months.

*See section 8 for schedule of tests during post treatment.

5.3.2.2 Economic endpoints (specific aim #2)

Trial-based economic analysis of menorrhagia are rare although the work of Hurskainen et al. and Cote et al. emphasize the importance of assessing direct

medical costs, patients costs, as well as productivity losses associated with this condition (24, 25):

1. Disease-specific direct medical costs [primary endpoint (specific aim#2)]: The economic analyses will focus on direct procedural and post-procedural costs of care (ablation procedures, hysterectomy), outpatient visits, and medication costs incurred by enrolled patients during 12 month follow-up to initial intervention. Assessment of resource utilization during this longer term follow-up period is important; it is plausible that a woman might undergo more than one procedure within 12 months such as a women randomized to medical therapy, who elects endometrial ablation or even hysterectomy as a second line therapy within the first year. The economic implications of this type of initial treatment failure may be significant.

Institutional administrative data will be used to track medical resource utilization and related billed expenditures for ablation procedures and office visit costs. Because of well-known discrepancies between billed charges and true resource use, we will value utilization using standard methods by grouping services into the Medicare Part A and B classification: Part A billed charges will be adjusted using hospital cost-to-charge ratios at the departmental level and wage indexes. Costs associated with Part B physician services will be proxied based on Medicare reimbursement rates. Resource utilization that occurs during longer term follow-up will also be valued in a similar manner. Average wholesale prices will be used to assess medication costs.

2. Patient and indirect costs: Costs associated with sanitary product use and indirect costs associated with lost work days or days with ability limitations will be assessed. Data on sanitary product use will be collected via patient survey at 3 month intervals (PBLAC; Appendix C). Utilization of sanitary projects will be valued using national average sales prices. Information on lost work days and days of limited capacity will be assessed via patient self report in the menstrual diaries (Appendix C). As others have done, lost work and limited ability days will be valued using the human capital approach, based on average hourly wage rates (25). We will undertake sensitivity analyses varying wage assumptions as well as the economic impact of days of limited ability.

3. Total costs will include direct medical, patient, and indirect costs and provide an estimate of costs from a societal perspective. These estimates will be presented undiscounted, as the study observation period is limited to 12 months follow-up to initial intervention.

4. If effectiveness of intervention is established via significant changes in health-related quality of life, we will also estimate the cost-effectiveness of treatment (defined as the incremental mean cost per quality-adjusted life year gained for ablation compared with medical therapy). Seven items from the SF-12® scores at the patient level will be used to estimate a preference-based measure of health using a publically available scoring algorithm (19). These preference-based measures of health (utilities) enable estimation of quality-adjusted life years gained with ablation compared with medical therapy (26).

5.4 Statistical Analysis:

Baseline demographic and clinical characteristics of this study will be reported. For normally distributed variables, the mean and standard deviation will be reported. For skewed data, the median and range with the corresponding quartiles will be

used. For binary variables, the proportions and their corresponding 95% CI will be reported.

Observed costs will be compared using Student's t-tests and nonparametric bootstrapped confidence intervals of mean differences. Individual observations will be weighted or the Lin method employed to account for censoring of costs (death or lost to follow-up), as appropriate. Generalized linear modeling techniques may also be used to assess the independent impact of treatment on economic outcomes to adjust for potentially confounding factors not accounted for in trial randomization. All analyses will be based on the intent-to-treat principle. All calculated p-values will be two-sided and p-values less than 0.05 will be considered statistically significant.

5.4.1 Power calculation and Statistical analysis for the Efficacy and Safety endpoints (specific aim #1)

The primary endpoint for specific aim #1 is the menstrual flow at 12 months measured using PBLAC scores. To take account of any possible imbalance in PBLAC scores at baseline between the two treatment arms, analysis of covariance (ANOCOVA) models will be used to compare the results at 12 months between the two treatment groups. However, given the lack of sufficient background information needed to calculate the power for an ANOCOVA model, an alternative approach was used (27).Based on the summary statistics reported in the literature for the PBLAC score, the distribution tends to be positively skewed. Therefore, the sample size calculation was based on the assumption that the data will be analyzed in on the natural logarithmic scale (Table 1). A study by Barrington et al in 2003 observed a 50% reduction in the mean PBLAC score at 6 months in patients who underwent a thermal ablation (mean (SD): baseline, 122 (74) and 6-months, 61 (99)(28) We are anticipating at least a 60% reduction at 12 months in

the proposed study. A study by Reid et al in 2005 observed a 30% reduction in the mean PBLAC at 6 months in patients who received medical therapy (14). We are anticipating at least a 40% reduction in the proposed study. In order to have 80% power to detect the anticipated differences, the study will need 98 patients per treatment group. Assuming a 20% drop out rate, a total of 240 patients will be recruited (120 per group).

Table(1): Sample size calculations for comparing the Mean PBLAC at 12 months between the two treatment groups, based on 80% power and type I error rate of 5%.

Anticipated Mean PBLAC Scores at 12 Months		Effect size†	Common within group standard deviation ‡	Estimated sample size per group *
Ablation group (M1)	Medical therapy group (M2)			
48.8 (assume 60% reduction at 12 months from baseline mean of 122)	73.2 (assume 40% reduction at 12 months from baseline mean of 122)	0.4055	0.66	43
			1	98
			1.6	250

†Effect size in the natural log scale is estimated by ln (M2/M1)
‡Common within group standard deviation in the natural log scale is estimated by CV=(SD/mean), both in the original scale.

- using the Barrrington et al mean (SD) at baseline, 0.66 = (74/122)
- using the Barrington et al mean (SD) at follow-up, 1.6 = (99/61)

*Estimated sample size per group = 1 + 16x(CV/effect size)^2, based on 80% power and type I error level of 5%.

(2/25/2011) Given the slower than anticipated recruitment, the study is being revised so that a total of 120 patients will be recruited, with the goal of having 50 patients per group complete the study. The table below summarizes the detectable effect size with 80% power for varying estimates of the common within group standard deviation. Assuming a common within group standard deviation of 1, the study will have 80% power to detect an effect size of 0.57. Assuming that the mean PBLAC score at 12 months in the ablation group is 48.8, this

effect size would equate to a mean PBLAC score at 12 months of 86.3 in the medical therapy group.

Sample size per group	Common within group standard deviation	Detectable effect size with 80% power
50	.66	0.38
	1	0.57
	1.6	0.91

Aim 1 will focus on differences between treatment groups for a variety of outcomes. Randomization and control of factors in the clinical trial should remove biases in the associations of treatment and outcomes. We will use stratification according to the age, BMI and parity. We will measure and adjust for other baseline factors related to the outcomes, such as the presence of fibroid, the presence of dysmenorrhea and the presence of anemia necessary to reduce noise associated with those factors. Measurements will be available from baseline, 3 month, 6 month, and 12-month contacts/visits. We will generate standard descriptive statistics for each time point. These include means, medians, standard deviations, ranges, and plots for continuous variables, and counts and percentages for categorical variables. Given that the distributions of some of the continuously scaled measures, such as PBLAC score, may be skewed, distributions will be examined for normality and skewness and transformations may be applied prior to formal analysis.

For each of the continuously scaled measures (PBLAC score, SF-12 composite score, hemoglobin, ferritin, and dysmenorrhea score), separate analysis of covariance (ANOCOVA) models will be fit to compare the 12 month measures between the two treatment groups, after adjusting for the baseline measures. This approach is unaffected by baseline imbalance in the measure between the two groups, and is

preferred over simply comparing change scores (post-pre) since this later approach does not control for baseline imbalance because to regression to the mean. Additional models will be fit to evaluate the results at the early time points.

Patient satisfaction (rated on a 5-point ordinal scale) will be compared between the two treatment groups using a Wilcoxon rank sum test. The presence of each premenstrual symptom, as reported in the PMS diary, will be tabulated and compared between the two groups using the chi-square test or Fisher's exact test, as appropriate.

To examine the effects of the treatments over time, we will use regression models with estimates obtained through generalized estimating equations (GEE) (according to the method developed by Liang and Zeger) to adjust for repeated measures on each subject. (29) These models will use main effects and interactions of time with treatment and other variables as predictors of the outcomes. If, for example, treatments are associated with differing hemoglobin levels over time, then the treatment x time interaction should be significant.

Kaplan-Meier plots will be used to evaluate the difference in the rate of treatment failure between the medical and the NovaSure® groups. The outcome [dependent variable (Y)] will be "treatment failure" which will include need for hysterectomy within 12 months after the procedure. For patients that have not had a treatment failure, their duration of follow-up will be calculated from the date of treatment to the date of their last follow-up (or death) within the 12 months following their treatment. A Cox proportional hazards model will be fit to evaluate the association between treatment and time to treatment failure. Additional models will be fit to evaluate the association after adjusting for any potential confounders. The strength of the association will be summarized by estimating hazard ratios and corresponding 95% confidence intervals (CI).

An additional analysis will be performed, based on the following definition of success/failure. The association between treatment group and success will be evaluated based on fitting a logistic regression model, with and without adjusting for

potential confounders. The strength of the association will be summarized by estimating odds ratios and corresponding 95% CIs. Subjects with incomplete PBLAC/QOL data at 6, 12 months will be classified as success, failure or lost to follow up according to evidence of performing another procedure (hysterectomy or ablation), and patients' self-reporting reporting of amenorrhea or bleeding (table 1). Otherwise subjects who complete the PBLAC chart and QOL diary at 12 months will be classified as success/failure based on their responses.

Table (1): Subjects with incomplete data at 12 months will be included or excluded in the primary end-point analysis:

Circumstance	Success	Failure	Lost to follow up
Any additional intervention to control bleeding before the 12th cycle (Including hysterectomy or ablation)		X	
PBLAC/QOL not completed at 12 months, subject reports no bleeding	X		
PBLAC/QOL not completed at 12 months, subject reports bleeding		X	
PBLAC/QOL completed at 6 months, reported amenorrhea, failed to follow-up at 12 months	X		
PBLAC/QOL completed at 6 months, and reported bleeding, failed to follow-up at 12 months			X
Failed to complete diaries at 6 and 12 months and failed to report on menstrual status.			X

6.0 Adverse Events:

An adverse event (AE) is any untoward medical occurrence in a study subject which does not necessarily have a causal relationship with the medicinal product. This includes any unfavorable and unintended sign (including an abnormal, clinically significant laboratory finding), symptom, or disease temporally associated with the use of a medicinal product, whether or not related to the medicinal product. It also includes any pre-existing condition that increases in intensity or severity or any new events after the patient has given informed consent.

6.1 Serious Adverse Events (SAE):

A serious adverse event is any untoward medical occurrence that results in one of the following:

- Death

- Life threatening illness or injury
- Permanent impairment of a body function or structure
- Inpatient hospitalization or prolongation of existing hospitalization;
- Medical or surgical intervention to prevent permanent impairment
- Fetal distress, fetal death, congenital abnormality, or birth defect

6.2 Unanticipated Adverse Device Effects (UADE):

Any serious adverse effect on health or safety, any life-threatening problem or death caused by, or associated with a device, if that effect, problem, or death was not previously identified in nature, severity, or degree of incidence in the application; or any other unanticipated serious problem associated with a device that relates to the rights, safety, or welfare of subjects.

6.2.1 Anticipated Adverse Effects of Endometrial Ablation:

The following complications are commonly reported post-operatively following an endometrial ablation procedure (please refer to the NovaSure® Instruction for Use located in Appendix A):

- Cramping/pelvic pain
- Nausea and vomiting
- Vaginal discharge
- Vaginal bleeding/spotting

With all endometrial ablation procedures, serious injury or death can occur. The following adverse effects could occur and have been reported in association with the use of the NovaSure® system:

- Thermal injury to adjacent tissue
- Perforation of the uterine wall
- Post-ablation tubal sterilization syndrome
- Air or gas embolism
- Complications with pregnancy (Note: Pregnancy following endometrial ablation is very dangerous for both the mother and the fetus)
- Infection or sepsis
- Complications leading to serious injury or death

6.2.2 Anticipated Adverse Drug Reactions of Non-Steroidal Anti-Inflammatory Drugs (Naproxen Sodium)

Serious Side effects include (please refer to Appendix B):

- heart attack
- stroke
- high blood pressure
- heart failure from body swelling (fluid retention)
- kidney problems including kidney failure
- bleeding and ulcers in the stomach and intestine
- low red blood cells (anemia)
- life-threatening skin reactions
- life-threatening allergic reactions
- liver problems including liver failure
- asthma attacks in people who have asthma

Other side effects include:

- stomach pain
- constipation
- diarrhea
- gas
- heartburn
- nausea
- vomiting
- dizziness

6.2.3 Anticipated Adverse Drug Reactions of Oral Contraceptive Pills (OCP):

Varying side effects can occur depending on the specific type of oral contraceptive that is prescribed. Risks of OCP include headaches, bloating, breast tenderness, blood clots, embolism and/or bleeding in your stomach.

6.3 Reporting of Adverse Events:

All adverse events (including SAE's and UADE's) will be recorded on the case report forms. Serious adverse events forms must be completed and faxed to NovaSure Clinical Affairs at 508-263-2965 within 3 days of knowledge of the event. Unanticipated adverse device effect and/or unanticipated adverse drug reaction forms must be completed and faxed to NovaSure Clinical Affairs within 24 hours of knowledge of the event.

6.4 Data and Safety Monitoring Plan

The Novasure device was FDA approved in 2002 with no major morbidity or mortality reported to date. In over 500 procedures performed at Mayo, only minor complications (cystitis, endometritis) have been noted. However, the principal investigator and Co-Investigator, Dr Mathew Hopkins will monitor the safety of the subjects and are available on call 24 hours, 7 days a week. A review of the complications data will be performed after the enrollment of 50 patients; any unexpected complication or major complication will prompt the PI to notify the IRB.

7.0 Ethical Considerations (Human Studies):

Informed consent will be obtained in the standard written form. Since the interventions are a component of routine clinical practice, we do not anticipate any ethical issues or risks beyond what is currently deemed as acceptable for standard practice. The risks of medical therapy include thrombo-embolic phenomena, upper gastro-intestinal bleeding and hypersensitivity reactions. Intra-operative events associated with NovaSure endometrial ablations are rare, but include cervical laceration and uterine perforation. Late complications include endometritis, cystitis and hematometra.

7.1 Study Duration

The participation of each subject in the study ends with the completion of the 12 month follow-up assessment. The enrollment phase of the study will be completed within 12 months following initiation. The entire study should be completed within 24 months of initiation.

8.0 Study Expectations and Limitations:

8.1 This study is an open label single center randomized clinical trial. Randomization will be done using a dynamic allocation process with stratification by age, parity and body mass index (BMI). This will be done to avoid any confounding effects of these variables

on our outcomes. We appreciate that the conduction of the study in one center might limit the external validity of our results and may be associated with some referral selection bias. In an effort to reduce the potential effect of referral and selection bias and to increase the external validity of the current study, we will extend the search of eligible patients to include "The Mayo Health System" which includes most of the community practice and family physicians in the surrounding community. Meanwhile, the inclusion of one center provides a chance of increasing the internal validity of the study measures and results. While masking (blinding) of the investigators and the patients to the treatment modality is not possible, the personnel responsible for allocation will be different from the physician who will perform the baseline assessment of the patients.

8.2 In assessing the efficacy and safety of GEA compared to low dose contraceptive pills as an initial step in the management of menorrhagia (specific aim #1) we will use various endpoints including the score of pictorial blood assessment chart (PBLAC), the change in the menstrual pattern, menorrhagia specific questionnaire, the change in hemoglobin and ferritin levels from baseline, the treatment satisfaction rate, the health-related quality of life assessment using SF-12, and the time to failure of treatment (time to hysterectomy or ablation). While the use of multiple endpoints may increase the possibility of a type I error (i.e. rejection of the null hypothesis while it is true; i.e, having a positive result by chance), a consistent positive result will strengthen the inference of the results. Nonetheless, we pre-selected the mean change in the PBLAC score as the primary endpoint *a priori*. From another point of view, there is currently no single outcome that can provide a complete evaluation of menorrhagia treatment. The choice of PBLAC scores as our primary endpoint depended on the high acceptability of this method by physicians and patients, as well as it being a reliable (precision) and valid (accuracy) surrogate for the assessment of menstrual blood loss (MBL). All these factors provide a strong ground for the use of PBLAC in clinical practice as well as in clinical research. While older quantitative (objective) methods for MBL using alkaline hematin, or total menstrual

fluid loss provide more accurate estimation of MBL, they are less convenient for the patients, and more expensive. (14, 18, 30-32)

8.3 For the evaluation of the cost associated with medical treatment and NovaSure (specific aim #2) we will include direct medical costs, costs to the patient, as well as indirect costs in an attempt to provide a comprehensive but surrogate measure of societal costs. A source of strength of our study is the detailed economic data collection at the patient level. We appreciate the presence of many alternate ways of estimation of indirect costs as well as the possibility of differences between institutions. We will use sensitivity analyses to evaluate the possible effects of alternative assumptions used in cost estimation.

8.4 In spite of these limitations, we expect this study to provide an answer to the use of NovaSure as an initial intervention in the management of menorrhagia in women who are not interested in preserving their fertility. Given the rigorous design methodology of this project we expect the inference derived from the results will be strong enough to alter and shape future clinical practice.

9.0 Literature Cited:

1. Nicholson WK, Ellison SA, Grason H, et al: Patterns of ambulatory care use for gynecologic conditions: a national study. Am J Obstet Gynecol 2001;184:523–30.
2. Oehler MK, Rees MC. Menorrhagia :an update. Acta Obstet Gynecol Scand 2003 May;82(5):405-22.
3. Bradley LD. Abnormal uterine bleeding. Nurse Pract. 2005 Oct;30(10):38-42, 45-9). (Albers JR, Hull SK, Wesley RM. Abnormal uterine bleeding. Am Fam Physician. 2004 Apr 15;69(8):1915-26.
4. Banu NS, Manyonda IT.Alternative medical and surgical options to hysterectomy. Best Pract Res Clin Obstet Gynaecol. 2005 Jun;19(3):431-49

5. Albers JR, Hull SK, Wesley RM. Abnormal uterine bleeding Am Fam Physician. 2004 Apr 15;69(8):1915-26.
6. ACOG Practice Bulletin. Management of Anovulatory Bleeding. Int J Gynaecol Obstet 2001; 73:263-71.
7. Cameron IT, Leask R, Kelly RW, Baird DT. The effects of danazol, mefenamic acid, norethisterone and a progesterone-impregnated coil on endometrial prostaglandin concentrations in women with menorrhagia. Prostaglandins 1987;34(1):99-110
8. Fraser IS, McCarron G. Randomized trial of 2 hormonal and 2 prostaglandin-inhibiting agents in women with a complaint of menorrhagia. Australian & New Zealand Journal of Obstetrics & Gynaecology 1991;31:66-70.
9. Roy S N, Bhattacharya S. Benefits and Risks of Pharmacological Agents Used for the Treatment of Menorrhagia. Drug Safety 2004; 27 (2):75-88.
10. Lethaby AE, Cooke I, Rees M. Progesterone/Progestogen releasing intrauterine systems for heavy menstrual bleeding. Cochrane Database Syst Rev. 2006;(1):CD002126
11. Iyer V, Farquhar C, Jepson R. Oral contraceptive pills for heavy menstrual bleeding. Cochrane Database Syst Rev. 2006;(1):CD000154.
12. Marjoribanks J, Lethaby A, Farquhar C. Surgery versus medical therapy for heavy menstrual bleeding. Cochrane Database Syst Rev. 2006;(1):CD003855
13. Hickey, M. Higham, J. Fraser, IS. Progestogens versus oestrogens and progestogens for irregular uterine bleeding associated with anovulation. Cochrane Database Syst Rev. 2006;(1):CD001895.
14. Reid PC, Virtanen-Kari S. Randomized comparative trial of the levonorgestrel intrauterine system and mefenamic acid for the treatment of idiopathic menorrhagia: a multiple analysis using total menstrual fluid loss, menstrual blood loss and pictorial blood loss assessment charts. BJOG. 2005 Aug;112(8):1121-5.
15. Bourdrez P, Bongers MY, Mol BW. Treatment of dysfunctional uterine bleeding: patient preferences for endometrial ablation, a levonorgestrel-releasing intrauterine device, or hysterectomy. Fertil Steril. 2004 Jul;82(1):160-6

16. Sowter MC, Lethaby A, Singla AA. Pre-operative endometrial thinning agents before endometrial destruction for heavy menstrual bleeding. Cochrane Database Syst Rev.2006;(1):CD001124
17. Lethaby A, Hickey M, Garry R. Endometrial destruction techniques for heavy menstrual bleeding. Cochrane Database Syst Rev. 2006(1):CD001501
18. Marjoribanks J, Lethaby A, Farquhar C. Surgery versus medical therapy for heavy menstrual bleeding. Cochrane Database Syst Rev. 2006;(1):CD003855.
19. Brazier J, Roberts J. Estimation of a preference-based measure of health from the SF-12. Med Care 2004;42; 851-859.
20. Brazier J, Roberts J, Deverill M. The estimation of a preference-based single index measure for health from the SF-36. J Health Econ 2002;21;271-292.
21. Higham JM, O'Brien PMS, Shaw RW. Assessment of menstrual blood loss using pictorial chart. Br J Obstet Gynaecol 1990; 97:734-9.
22. Belsey EM, Machin D, d'Arcangues C. The analysis of vaginal bleeding patterns induced by fertility regulating methods. Contraception 1986;34:253-60.
23. Belsey EM, Pinol APY, Task Force on Long-Acting Systemic Agents for Fertility Regulation. Menstrual Bleeding Patterns in the untreated Women. Contraception 1997; 55:57-65.
24. Hurskainen R, Teperi J, Rissanen P, Alto A-M, Grenman S, Kivelä A, Kujansuu E, Vuorma S, Yliskoski M, Paavonen J. Clinical outcomes and costs with the levonorgestrel-releasing intrauterine system or hysterectomy for treatment of menorrhagia: randomized trial 5-year follow-up. *JAMA. 2004;*291:1456-1463.
25. Cote I, Jacobs J, Cumming D. Work loss associated with increased menstrual loss in the United States. Obstet Gynecol 2002; 100: 683-7.
26. Gold MR, Siegel JE, Russell LB, Weinstein MA (eds.) *Cost effectiveness in health and medicine*. New York: Oxford University Press, 1996.
27. Dallal GE. The Little Handbook of Statistical Practice. Online 2/17/2006. Accessed 12/14/2006. <http://www.tufts.edu/~gdallal/LHSP.HTM >
28. Barrington JW, Arukalaivanan AS, Abdel-Fattah M. Comparison between the levonorgestrel intrauterine system (LNG-IUS) and therma ablation in the treatment of

menorrhagia. Euro J Obtet Gynecol Reprod Biol 2003; 108:72-4. Liang KY, Zeger SL. Regression analysis for correlated data. Annu Rev Public Health. 1993;14:43-68.

29. Liang KY, Zeger SL. Regression analysis for correlated data. Annu Rev Public Health. 1993;14:43-68.
30. Hallberg L, Nilsson L. Determination of menstrual blood loss. Scand J Clin Lab Invest 1964: 16: 244-8.
31. Shaw ST Jr, Aaronson DE, Moyer DL. Quantitation of menstrual blood loss--further evaluation of the alkaline hematin method. Contraception. 1972 Jun;5(6):497-513
32. Reid PC, Coker A, Coltart R. Assessment of menstrual blood loss using a pictorial chart: a validation study.Br J Obstet Gynaecol 2000; 107:320-2.

10.0 Observations: (Note: All case report forms include fields for patient ID, patient initials, comments, and investigator signature)

Event	Clinical evaluation	Clinical forms	Subject forms	Tests
Pre-enrollment (-1 months)	1. History and physical examination.<br>2. Pelvic examination.<br>3. Office hysteroscopy<br>4. Pipelle biopsy<br>5. Pap Test	1.Screening log	1. PBLAC*<br>2. Dysmenorrhea VAS<br>3. PMS diary<br>4. SF-12<br>5. Menorrhagia QOL	
Enrollment (0 month)		1. Enrollment<br>2. Preoperative evaluation<br>3. Randomization	1. PBLAC*<br>2. Dysmenorrhea VAS<br>3. PMS diary<br>4. SF-12<br>5. Menorrhagia QOL	1. Hemoglobin<br>2. Ferritin<br>3. FSH<br>4. Serum HCG (if indicated)
Procedure		1.Operative evaluation		
3 month follow up visit	Pelvic examination if clinically indicated	Documentation of additional procedure	1. PBLAC*<br>2. Dysmenorrhea VAS<br>3. PMS diary<br>4. SF-12<br>5. Menorrhagia QOL	1. Hemoglobin<br>2. Ferritin
6 month follow up visit	Pelvic examination if clinically indicated	Documentation of additional procedure	1. PBLAC*<br>2. Dysmenorrhea VAS<br>3. PMS diary<br>4. SF-12<br>5. Menorrhagia QOL	
9 month phone contact		Documentation of additional procedure	Mail out;<br>1. PBLAC<br>2. Dysmenorrhea VAS<br>3. PMS diary<br>4. SF-12<br>5. Menorrhagia QOL	
12 month follow up visit	Pelvic examination if clinically indicated		Return 9 month questionnaires	1. Hemoglobin<br>2. Ferritin<br>3. FSH (ablation arm only)

Patient Enrollment:

Year of Birth	Enter year of birth as YYYY
Date of Enrollment	Date patient agreed to participate and signed Informed consent documentation
Height	Report in centimeter
Weight	Report in kilogram
Body Mass Index	Report in kg/cm^2
The patient must:<br>1. Be an adult female who is premenopausal and for whom childbearing is complete<br>2. Subjective symptom of excessive menstrual loss<br>3. Have an anatomically normal uterine cavity length (≥ 4cm) with a sound measurement of ≤10cm documented by sonohystogram or hysteroscopy in the preceding 6 months<br>4. Have at least one normal Pap Test within past 3 years and no unexplained abnormal Pap Tests within 6 months of the procedure<br>5. Prior history of permanent sterilization or use of reliable non-hormonal contraception during the 14 month study period<br>6. Agree to use barrier birth control methods for the duration of the study.<br>7. Freely agree to participate in the study including all study related procedures and evaluations, and document this agreement by signing the informed consent document	All must be confirmed for enrollment

Selection Criteria:

The patient must not:<br>1. Be pregnant or desire for future childbearing<br>2. Have uterine sound measurement greater than 10cm<br>3. Have active lower genital infection at the time of procedure<br>4. Have active urinary tract infection at the time of procedure<br>5. Have active pelvic inflammatory disease (PID) or recurrent chronic PID<br>6. Have endometrial neoplasia, determined by endometrial biopsy taken within 6 months of study entry<br>7. Current or past history of cervical or endometrial cancer<br>8. Submucous leiomyoma greater than 2cm or cavity distorting leiomyoma<br>9. Have history of myomectomy or classical cesarean section<br>10. Have had a previous endometrial ablation procedure<br>11. Oral hormonal treatment in the preceding 3 months, hormone releasing intrauterine contraceptive in the preceding month, or injectable hormone treatment in the preceding 12 months<br>12. Have contraindications to oral contraceptive pill therapy;<br>-current or past history of thrombophlebitis or thrombo-embolic disorder<br>-cerebrovascular or coronary artery disease<br>-thrombogenic valvulopathies<br>-diabetes with vascular involvement<br>-uncontrolled hypertension<br>-known or suspected sarcoma of the breast<br>-cholestatic jaundice of pregnancy or jaundice with prior pill use<br>-hepatic adenomas or carcinomas or active liver disease<br>-hypersensitivity to Nordette components (Ethinyl estradiol and levonorgestrel)<br>13. Have contraindications to non-steroidal anti-inflammatory agents;<br>-hypersensitivity to naproxen<br>-rhinitis, urticaria, asthma, or allergic reactions to aspirin or other anti-inflammatory agents<br>-history of acute gastro-intestinal bleeding<br>14. History of a coagulopathy or endocrinopathy<br>15. Inability to follow up at 12 months	The only allowable response for enrollment is “0 none”<br><br>See inclusion criteria regarding oral contraceptives

Gynecological Diagnostic procedure:

GYN diagnostic procedures (date)<br>Hysteroscopy<br>Sonohysterogram	Report date of any diagnostic procedure used to qualify patient for enrollment<br>At least one must be performed within six months of study entry
Endometrial biopsy	Report date
Pap Test	Report date
Serum Pregnancy Test (date)<br>(1) Negative<br>(2) Not done, prior sterilization<br>(3) Positive	If positive, patient may not be enrolled
Medications:<br>Anticoagulant<br>Hormone<br>Analgesic*	Report medication, dose, frequency, duration, and indication for only those medication classes listed<br>Enter only medications patient is currently taking<br>*May not be on NSAIDs prior to study enrollment

Patient Evaluation (Pre-op, 3, 6 month, and 12 month visits)

Date of Evaluation	
Pelvic Exam – if clinically indicated<br>Vagina<br>Cervix<br>Uterus<br>Other	For each anatomical area report any abnormal findings or report 'none'
Uterine sound measurement	Interim value, final will be recorded immediately prior to procedure
Diary Data	Taken from patient diary
Length of cycle	Will use length of cycle-like symptoms (pain/cramps) for patients with amenorrhea<br>For those with no symptoms or bleeding, will report as 0 days
Pad count<br>Sum	
Diary score	
Pelvic pain score<br>Sum (total of monthly pain scores)<br>Maximum (highest single pain score)	
PMS symptom score	Premenstrual Symptoms: This includes depression, anger, irritability, moodiness, anxiety confusion, social withdrawal, breast tenderness, abdominal bloating, headache and swelling of extremities and occurs during the days just before the start of a period.

Procedure Observations

Date of admission	
Date of procedure	
Start date of last menstrual cycle	Last cycle immediately prior to procedure
Anesthesia (primary)<br>(1) Cervical Block<br>(2) General Anesthesia<br>(3) Epidural Anesthesia<br>(4) IV sedation	
Pain Control prescribed<br>Pre-operative<br>Intra-operative<br>Post-operative	Report medication and dose, regimen
Uterine sound measurement	Report in cm
Uterine position:<br>(1) anteverted<br>(2) mid-position<br>(3) retroverted	
Procedures Performed Prior to Ablation<br>(1) Suction curettage<br>(2) Sharp curettage<br>(3) Hysteroscopy<br>(4) Other – please describe	
Device Serial Number	
Procedure Start Time (Novasure device inserted)	Report on 24 hour clock
Uterine Cavity Length	Report in cm
Uterine Cavity Width	Report in cm
Treatment Time	Report in sec
Power	Report in watts
Procedure End Time	Time of device removal for completed procedure or time of procedure interruption for incomplete procedures. Report on 24 hour clock.
Procedure interruption/restart	Describe problem and when it occurred. If procedure is restarted then Form Q (restart form) must be filled out. A new form Q must be filled out every time the procedure is restarted.
Adverse Clinical Experiences During Procedure;<br>-uterine perforation<br>-cervical laceration	Report and describe on Adverse Experiences form

-severe pelvic pain, nausea<br>-hemorrhage	
Device complications during the procedure	
Complications Resulting From Procedure<br>-urinary tract infection<br>-endometritis<br>-hematometra<br>-pelvic inflammatory disease<br>-pelvic abscess<br>-chronic pelvic pain (>3 months post-procedure)	Report and describe on Adverse Experiences form
Date of discharge	
Adverse reaction to oral contraceptive pills<br>Document any of the following:<br>-nausea<br>-vomiting<br>-abdominal bloating<br>-breakthrough vaginal bleeding<br>-edema/fluid retention<br>-mastalgia (breast pain)<br>-weight gain (in kilograms)<br>-mood changes<br>-acne<br>-intolerance to contact lenses<br>-confirmed or suspected diagnosis of thrombo-embolic phenomena	
Adverse reaction to Naproxen®<br>-indigestion<br>-nausea<br>-epigastric pain<br>-vomiting<br>-constipation<br>-diarrhea<br>-headache<br>-dizziness or drowsiness<br>-irritability<br>-vision or hearing disturbances<br>-skin rash<br>-shortness of breath/wheezing	

6 & 12 Month Follow-up Visit

(see section above)

Patient Diary

Cycle start date	
Cycle end date	
Pad count<br>Sum	Used for cycles 1-5 and 7-11
Diary Score	Used for cycles 6 and 12
Pelvic Pain Score<br>Sum<br>Max	On a scale of 0-10 report the worst pelvic pain/cramping experienced for each day of menstruation
Pain Medication Usage<br>Sum<br>Max	
PMS symptom score<br>(0) none<br>(1) mild<br>(2) moderate<br>(3) severe	

Adverse Event – Use for serious adverse events and unanticipated adverse device effects

Complete and fax to Cytyc Surgical at 508-263-2965 per the protocol when recording serious or unanticipated adverse events	
Date of observation	Date patient was seen by investigator and event was observed
Description of adverse event	
Started	Report of date and time the adverse event first began
Resolved	Report of date adverse event resolved, or marked as ongoing
Severity<br>(1) Mild<br>(2) Moderate<br>(3) Severe	
Device Related<br>(1) No<br>(2) Yes<br>(3) Undetermined	
Description of management	Detailed description of steps taken to manage adverse event with outcome or expected outcome defined

Additional Procedure – Additional Gynecological procedures performed

Date of procedure	Please also indicate if this procedure was done at MCR or elsewhere
Description of procedure	
Reason for procedure	
Outcome of procedure	

Withdrawal/Termination

Date of withdrawal/termination	Effective date subject withdrew or was terminated from the study
Reason for discontinuation<br>(1) Patient request<br>(2) Lost to follow-up<br>(3) Death<br>(4) Additional Procedure<br>(5) Other	For each reason, provide detail in the comments section of the form<br>For lost to follow-up document attempts to contact patient<br>For death, provide information regarding cause of death<br>For additional procedures, report on F71 (Additional Procedures Form)

11.0 Appendices

Appendix A: NovaSure® Instructions for Use

Appendix B: Naproxen Sodium Tablets USP

Appendix C: Study Questionnaires:

Menstrual Symptom Cycle Diary[1] and the Pictorial Blood Loss Assessment Chart [2]

Part (I).Menstrual Diary for the previous month:

Month: --/---- (mm/yyyy)

Please complete the self-assessment by rating the degree of severity (none to severe) you are experiencing for each symptom listed below at the end of each day of the calendar month. (Record: non=0; mild=1, moderate=2; severe=3)

Day of the month	1	2	3	4	5	6	7	8	9	10	11	12	13	14	15	16	17	18	19	20	21	22	23	24	25	26	27	28	29	30	31
Bleeding																															
Pain																															
Irritability																															
Mood Swing																															
Nervous tension																															
Anxiety																															
Depression																															
Feeling out of control																															
Poor coordination																															
Confusion																															
Insomnia																															
Crying																															
Fatigue																															
Food craving																															
Breast tenderness																															
Swelling																															
Cramps																															

[1] Freeman EW, DeRubeis RJ, Rickels K. Reliability and validity of a daily diary for premenstrual syndrome. Psychiatry Res. 1996;65(2):97-106.

[2] Higham JM, O'Brien PMS, Shaw RW. Assessment of menstrual blood loss using a pictorial chart. Br J Obstet Gynaecol 1990; 97: 734–9.

Day of the month	1	2	3	4	5	6	7	8	9	10	11	12	13	14	15	16	17	18	19	20	21	22	23	24	25	26	27	28	29	30	31
Aches																															
Headache																															
Please mark any day that you could not go to work																															
Please mark any day that you have not been able to carry on your usual daily activity																															

Part (I1). Pictorial Blood Loss Assessment Chart for the previous month:

The previous period started: --/--/---- (mm/dd/yyyy)

Sanitary Pads/ Tampons			
Sanitary Pads (please check one)	a) Regular	b) Super absorbency	c) Ultra thin
Tampons (please check one)	a) Regular	b) Super absorbency	c) Ultra thin

Days	D1	D2	D3	D4	D5	D6	D7	D8	D9	D10	D11	D12			
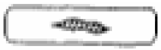															
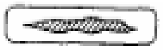															
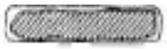															
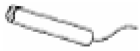															
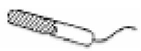															
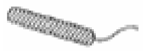															
Small blood clots (small coin)															
Large blood clots (> quarter)															
Number of menstrual accidents															

Instructions:

1. Indicate the type and number of sanitary pads or tampons that you used on each day of your last menstrual period.
2. Choose the picture that most closely represents the degree of staining of each pad or tampon used.
3. Indicate the presence and size of blood clots passed during your last menstrual period.
4. Indicate the occurrence of any menstrual accident during your last menstrual period (underwear soilage, seat or bed sheet soilage) in spite of protection.
5. If both sanitary pads and tampons are used, please indicate the type and number of both of them

On a scale from 1 to 10, how would you rate the pain during your menstrual period?	(no pain) ├────────┤ (worst pain ever)<br>0 1 2 3 4 5 6 7 8 9 10
